# In Vitro Bioaccessibility and Speciation of Toxic and Nutritional Trace Elements in Brazil Nuts

**DOI:** 10.3390/ijms26178312

**Published:** 2025-08-27

**Authors:** Astrid Barkleit, Jiyoung Eum, Diana Walther, Daniel Butscher, Sebastian Friedrich, Katharina Müller, Jerome Kretzschmar

**Affiliations:** 1 Helmholtz-Zentrum Dresden–Rossendorf, Institute of Resource Ecology, 01328 Dresden, Germany; j.eum@hzdr.de (J.E.); d.butscher@hzdr.de (D.B.); s.friedrich@hzdr.de (S.F.); k.mueller@hzdr.de (K.M.); j.kretzschmar@hzdr.de (J.K.); 2 VKTA—Radiation Protection, Analytics & Disposal Rossendorf Inc., 01328 Dresden, Germany; diana.walther@vkta.de

**Keywords:** Brazil nut flour, selenium, radium, alkaline earth metal, alpha/gamma spectrometry, luminescence spectroscopy, nuclear magnetic resonance spectroscopy, decorporation agents, aminocarboxylate, hydroxypyridinone

## Abstract

Brazil nuts (*Bertholletia excelsa*), mainly from the Amazon, are notable for their exceptionally high selenium (Se) content and are widely consumed as a natural dietary supplement. They also contain potentially harmful elements, including barium (Ba), and exhibit an unusual capacity to accumulate radioactive radium (Ra). In this study, we quantified the concentrations of Se, Ba, strontium (Sr), lanthanum (La), europium (Eu), and the radionuclides ^226^Ra and ^228^Ra, and assessed their in vitro bioaccessibility—data largely unavailable for these elements to date. Se was highly bioaccessible (≈85%), whereas Ba and Ra, both chemo- and/or radiotoxic, exhibited low bioaccessibility (≈2% each). Nuclear magnetic resonance (NMR) spectroscopy revealed Se to occur predominantly as selenomethionine (SeMet), alongside phytate, amino acids, peptides, and other polar low-molecular-weight compounds. The influence of Brazil nut flour (BNF) on Eu(III) speciation in simulated gastrointestinal fluids, and the effect of chelating agents such as ethylenediaminetetraacetic acid (EDTA), diethylenetriaminepentaacetic acid (DTPA), and the hydroxypyridinone 3,4,3-LI(1,2-HOPO) were investigated using time-resolved laser-induced fluorescence spectroscopy (TRLFS). Results indicate that the food matrix has only a minor impact on the decorporation efficacy of these chelators. These findings provide novel insights into the bioaccessibility and chemical speciation of nutritionally and toxicologically relevant elements in Brazil nuts.

## 1. Introduction

Brazil nuts originate almost exclusively from the Amazon region, typically containing 60–70% of lipids, 14–17% of proteins, and 10–16% of carbohydrates [1,2,3]. Brazil nuts are a very common natural food supplement due to their extremely high selenium (Se) content [2,3,4]. They are also rich in organic nutrients like protein, fiber, thiamin, niacin, pyridoxine, vitamin E, and essential minerals such as calcium (Ca), magnesium (Mg), iron (Fe), potassium (K), copper (Cu), zinc (Zn) and phosphorus (P) [2,3]. Se in particular, in addition to some organic micronutrients like phytosterols, flavonoids, or polyphenols are known to have healthy effects due to their antioxidant and anticarcinogenic properties [2,3,4,5].

Selenium is an essential trace element, being a part of about 25 selenoproteins in mammals, which are crucial to several metabolic functions like oxidative stress balance or redox signaling [2,4]. However, the health effects of Se are confined to a narrow intake range. The recommended dietary allowance for adults is about 55–70 µg/day [4,6], and the tolerable upper intake level to prevent harmful effects, recommended by the European Food Safety Authority (EFSA), is 255 µg/day [7]. An excess of Se intake can lead to several toxic effects (selenosis) like hair loss, broken and sloughing nails and even cardiovascular pathology, endocrine dysfunction, and cancer [2,4]. The Se concentrations in Brazil nuts have therefore been measured and published very extensively so far [4,8,9,10,11,12,13,14,15,16,17,18,19,20,21,22,23,24].

Brazil nuts can also accumulate non-essential, potential toxic heavy metals from the group of alkaline earth metals like barium (Ba), strontium (Sr), and radium (Ra), a naturally occurring radioactive metal [2,6,25], and rare earth elements (REEs), including the group of lanthanides (Ln). These metals can substitute for Ca in minerals both in nature and within the human body. Ba and Ra are primarily accumulated in the skeletal system (approximately 90%), as well as in teeth, heart, lung, kidney, and liver, causing toxic effects like cardiac and renal failure or pulmonary edema [26,27,28]. In contrast, there is no evidence of toxic effects of Sr in adults [2]. The most common radioactive isotopes of Ra are the alpha emitter ^226^Ra and the beta emitter ^228^Ra. Both ionizing radiation types cause cell death, DNA damage, and lead to various cancer types via oral exposure [28].

Threshold levels for Ba intake in drinking water, as defined by the World Health Organization (WHO), are 1.3 mg/L (no corresponding data available for Sr) [29]. The U.S. Environmental Protection Agency (EPA) specifies oral reference doses (RfD) of 0.6 mg/kg body weight (BW)/day for Sr and 0.2 mg/kgBW/day for Ba. In addition, the Agency for Toxic Substances and Disease Registry (ATSDR) defines minimal risk levels (MRL) of 2.0 mg/kgBW/day for Sr and 0.2 mg/kgBW/day for Ba [26,30,31,32,33]. For Ra, no acceptable intake level has been established due to its radioactivity. Instead, the general dose limit for ionizing radiation for the public is set at 1 mSv/year. Furthermore, conversion factors, i.e., effective dose coefficients (Sv/Bq), for adults are given as 2.8 × 10^−7^ for ^226^Ra and 6.9 × 10^−7^ for ^228^Ra [25,34]. While Ba and Sr concentrations in Brazil nuts are still relatively common in the literature [18,19,20,21,22,23,24,35,36,37,38,39], data for Ra are scarce [12,25,38,39,40,41,42].

REEs have gained increasing importance due to their extensive use in modern technologies. This has led to a marked increase in exploitation and production, consequently raising concerns about potential risks to human health [43,44,45,46,47]. REEs accumulate from soil into roots and shoots of plants and can ultimately enter the human body via the food chain [48]. Their cytotoxic effects on human cell lines include DNA damage and cell death [43,49,50,51,52,53]. However, due to the complexity of the interactions within biological and environmental systems, the behavior of REEs remains insufficiently understood [46,47,54]. To date, no threshold levels for REEs have been established with the exception of a recent Chinese study recommending a safe daily intake of less than 70 µg/kgBW/day [44,45]. Only one study has so far investigated REE concentrations in Brazil nuts [21].

Determining the mere concentrations of the elements in the nuts is not sufficient to predict health effects. Since the absorption after oral consumption and digestion might be incomplete, the fractions which are absorbed during the digestion process into the body, the bioavailability, provides crucial information. More precisely, bioaccessibility is the proportion of an element or compound that is released from the food matrix during digestion and is accessible for absorption in the small intestine, while bioavailability indicates the proportion that is absorbed in the body [55,56]. Despite the importance, there is very little data on bioavailability or bioaccessibility from nuts for Se, Sr and Ba [8,14,20,57]. For Ln and Ra, no data are published so far.

In this study, we investigated the concentrations of selected trace elements and heavy metals in Brazil nuts, focusing on Se, the alkaline earth metals Ba, Sr, and the radionuclides (RNs) ^226^Ra and ^228^Ra, as well as lanthanum (La) and europium (Eu) as light (La) and intermediate (Eu) representatives of REEs. In addition, we assessed their respective bioaccessibilities during the human digestion process. The digestion was simulated in vitro, following the unified bioaccessibility method (UBM) developed by the Bioaccessibility Research Group of Europe (BARGE) [58,59,60]. Element concentrations were determined using inductively coupled plasma mass spectrometry (ICP-MS), and gamma or alpha spectrometry was used to determine RN concentrations, respectively.

An important point influencing bioavailability or toxicity of an element is its speciation, i.e., its chemical state in the system [61]. Recent studies identified Se to occur in Brazil nuts usually in organic form as the amino acid selenomethionine (SeMet), which is highly soluble and well absorbed by the human body [4,11,17,62,63]. Ba seems to form low soluble compounds such as BaSO_4_ or, owing to the chemical similarity of sulfur and selenium, as BaSeO_4_ [6,36]. For a better process understanding on the molecular level, we determined the speciation of selected elements after simulated digestion, namely Eu by time-resolved laser-induced fluorescence spectroscopy (TRLFS) and Se by nuclear magnetic resonance (NMR). By means of the latter, several organic compounds from Brazil nuts soluble in diluted aqueous hydrochloric acid (HCl, mimicking stomach digestion conditions) could also be identified.

In cases of occupational ingestion of toxic elements, an effective decorporation treatment is crucial to promote the elimination of the toxin. Special decorporation agents (DAs) are already in use or have been newly developed, particularly for highly radio- and chemotoxic actinides [64,65,66]. Since Eu from the Ln series has chemical properties comparable to some trivalent actinides, namely americium or curium, it is often used as a non-radioactive surrogate for chemical investigations like its complexation behavior with DAs [66]. Therefore, we investigated the influence of food matrix on the speciation of Eu in absence and presence of selected DAs like the aminocarboxylates ethylenediaminetetraacetic acid (EDTA) and diethylenetriaminepentaacetic acid (DTPA), which are commonly in use [67], as well as the newly developed chelator from the hydroxypyridinone group, 3,4,3-LI(1,2-HOPO), which is currently in clinical trials [68,69,70].

## 2. Results and Discussion

### 2.1. Element and Radionuclide Analysis in Brazil Nuts

We quantitatively analyzed the content of the trace element Se and the heavy metals Sr, Ba, La, and Eu as well as the RNs ^226^Ra and ^228^Ra in whole Brazil nuts and in defatted Brazil nut flour (BNF), both commercially available samples. Brazil nuts contain about 60–70% oil or lipids [2,3,15,22,23,24]. However, it has been shown that nearly all Se, Ba, and Sr is present in the defatted part and not in the lipid fraction [15,22,23,24].

In Table 1, our results for whole Brazil nuts and BNF are compared with the literature values, if available. All determined element or RN concentrations are in the range of published data. The Ba concentration is the highest, followed by Sr, significantly less Se, and traces of Eu and La. The observed variability is substantial, as Brazil nuts constitute a natural product. Their elemental concentrations are primarily determined by soil composition and a range of environmental parameters, including climatic conditions, geochemical mobility, and the accumulation and distribution processes within the plants. These concentrations are further modulated by complex chemical, physical, and biological processes [4,6,11,46,47,71]. The content of ^226^Ra and ^228^Ra fits also in the range of published data [25].

The ratios between element concentrations in the defatted flour are three to four times as high as in whole nuts, which, with an assumed lipid content of approximately 60–70%, confirms that the elements are mainly present in the lipid-free fraction.

### 2.2. Bioaccessibility of Selected Elements and Radionuclides in Brazil Nut Flour

**Figure 1:**
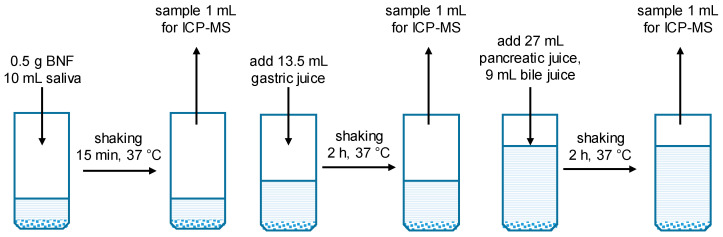
Workflow of the in vitro digestion procedure of Brazil nut flour (BNF).

Since the bioavailability or bioaccessibility provides a more accurate assessment of potential health effects than total element content alone, we have simulated the digestion process in vitro to obtain the bioaccessibility of the selected elements from Brazil nuts. We carried out the experiments following the UBM protocol, which is based on human physiology [58,59,60]. We mixed BNF stepwise with artificial saliva, gastric juice, pancreatic and bile juice, and stirred at body temperature for the necessary time (see Figure 1). The pH was measured at all digestion steps. The most important change during the digestion process was observed after the stomach digestion step. The pH of the stomach mixture (saliva + gastric juice) of about 1.5 has raised up to 3.5–4 in presence of BNF. This is in line with the human physiology of digestion. The pH increases up to 5 after food intake. Then, the acidity is upregulated by secretion of HCl for optimal enzyme activities, which is pH 4–6 for gastric lipase and pH 2–4 for pepsin. Therefore, it is recommended to set the pH to ~3 in the gastric phase for in vitro digestion investigations [56,72].

**Figure 2:**
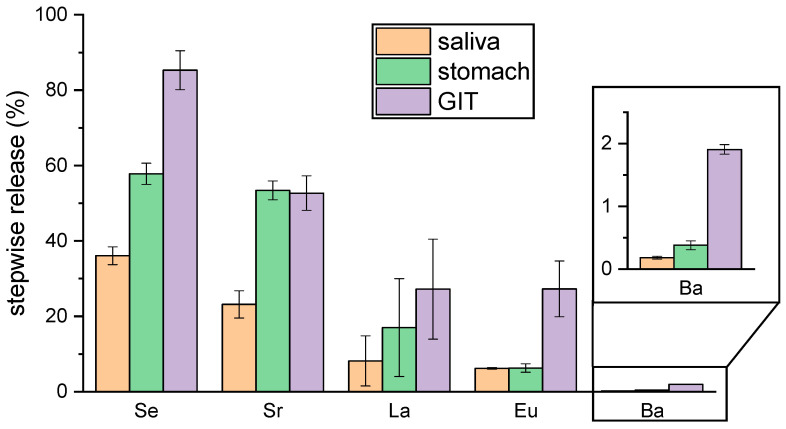
Stepwise release of elements by digestion simulation (GIT = complete gastrointestinal tract mixture).

Samples were taken for analysis before and after every digestion step. The results of the digestion simulation are summarized in Table 2 and Figure 2.

All elements show a stepwise release from BNF starting with saliva followed by the stomach mixture and culminating in the complete digestive mixture (gastrointestinal tract fluid, GIT) (Table 2 and Figure 2). This might be due to the increasing reaction time and/or the gradual rise in enzyme number and concentration upon stepwise addition of the biofluids. Both factors promote the dissolution of BNF and may thereby facilitate the release of the investigated elements.

While bioaccessibility and bioavailability data for Se from Brazil nuts are already available [8,14], corresponding data for the other metals investigated are scarce—limited to a single study for Sr and Ba [20]—and, to our knowledge, entirely lacking for La, Eu, and Ra.

The very high bioaccessibility of Se (approximately 85%) is comparable with published data for Brazil nuts [8,14] as well as other nuts and seeds [57]. Although most studies have examined whole Brazil nuts, they found similar results to our investigation of defatted BNF. This can be explained by the fact that almost all Se species present in Brazil nuts, especially the methionine- and cysteine (Cys)-bound ones, form SeMet and SeCys, and are highly water soluble and bioavailable [4,11,17,62,63]. The strongly deviating value of only 19% Se bioavailability published by Moreda-Pineiro et al. [20] might be due to different digestion simulation procedures. In this study, the authors performed a dialyzability procedure which could cause a different behavior of Se, resulting in different bioavailability.

Assuming the consumption of one Brazil nut per day (~5 g) [2,73] and considering a Se content range of 1–55 µg/g (Table 1) with 85% bioaccessibility, this corresponds to an estimated Se intake of approximately 4–235 µg/day. This value remains below the European tolerable upper intake level of 255 µg/day for avoiding harmful effects [7].

With 2%, Ba has the lowest bioaccessibility. One explanation for this could be that Ba is present in Brazil nuts as the extremely insoluble BaSO_4_ [6,36], as concluded from fractionation [36] or XRF- (X-ray fluorescence) mapping [6], merely detecting Ba and S simultaneously in the same fraction or at similar positions, respectively, not necessarily proving the presence of BaSO_4_ as an originating compound. However, it has been shown that at 37 °C after 3 h, more than 95% Ba has been dissolved from Brazil nuts with 0.1 M HCl [74]. Our own dissolution experiments confirm this result (see Figure S1, Supplementary Materials). While at room temperature in 0.01 M HCl, almost no Ba was extracted from BNF and only about 20% from whole Brazil nuts; we observed, using 0.1 or 1 M HCl, approximately 60% of Ba was extracted from BNF after 6 h, and nearly 80% after 15 h. From whole Brazil nuts, more than 80% of Ba was extracted after 6 h and approximately 100% after 15 h, respectively. These findings contradict the assumption of BaSO_4_ being the prevailing chemical form of Ba in Brazil nuts as BaSO_4_ is insoluble in HCl solutions [75]. Therefore, other binding forms or processes seem to be responsible for Ba’s low bioaccessibility. In consideration of the NMR analyses of the HCl soluble fraction (see Section 2.3), it is quite likely that Ba is associated with anionic sites present in the numerous and versatile organic compounds (e.g., carboxylates), among which we consider phytate (1,2,3,4,5,6 hexakis (di-hydrogen phosphate) myo-inositol) the most probable. The latter, being rich in phosphate groups, is commonly known to strongly bind metal ions and even reduce their uptake (bioavailability) [76,77,78,79]. In accordance with the observation, under very acidic conditions, upon protonation of phytate’s phosphate groups, the affinity for metal ion coordination is reduced, releasing the formerly bound metal ions. For sufficiently high pH (depending on the chemical environment, such as post-gastric sections), metal ion sequestration is effective, hence limiting uptake of dietary minerals into the body. However, the chemical binding form of Ba in Brazil nuts still remains unclear.

Assuming again a daily consumption of one Brazil nut (5 g), a maximum Ba content of approximately 2500 µg/g (Table 1), and 2% bioaccessibility, the resulting daily Ba intake would be about 50 µg/day. This is well below the reference dose (RfD) established by the U.S. EPA and the minimal risk level (MRL) set by the ATSDR of 0.2 mg/kgBW/day [30,31].

Ra shows bioaccessibility similar to that of Ba. This was to be expected, as it is known that Ra and Ba have very similar chemical behaviors. Furthermore, a recent study in the field of radiopharmaceutical research combining in vitro binding experiments with in vivo biodistribution experiments with mice demonstrated that treatment of Zn-phytate could significantly reduce the absorption of Ra from the intestine into the blood [80].

Using the effective dose coefficients (Sv/Bq) of 2.8 × 10^−7^ for ^226^Ra and 6.9 × 10^−7^ for ^228^Ra [25,34], the measured activities (Bq) can be converted into effective doses (Sv). For a daily consumption of one Brazil nut (5 g), and a bioaccessibility of 2%, the resulting effective dose is approximately 0.7 µSv/year for ^226^Ra. Assuming an approximately similar activity of ^228^Ra in Brazil nuts (Table 1) [25], this would contribute an additional effective dose of about 1.7 µSv/year, yielding a total effective dose of approximately 2.4 µSv/year (based on ^226^Ra and ^228^Ra). In their review, Koeder and Keller estimated that long-term consumption of one Brazil nut per day results in an effective dose of approximately 100 µSv/year, based on measured Ra activities of Brazil nuts [25]. When adjusted for the 2% bioaccessibility determined in our study, our results show excellent agreement with this estimate.

Both Ba and Ra are excreted predominantly via feces, and to a lesser extent via urine (feces-to-urine ratio ~9 for Ba and ~36 for Ra [81,82]), with approximately 80–90% eliminated within 10 days to 2 weeks after incorporation [25,26,27,28,81,82]. A residual fraction, however, remains sequestered in the skeleton [25,27,28]. Thus, despite their limited bioaccessibility and relatively short biological half-life, Ba and Ra remain toxicologically relevant due to their skeletal accumulation.

In contrast, the lighter alkaline earth metal Sr showed a much higher bioaccessibility with 52%. It is hence assumed that Sr’s binding behavior in Brazil nuts is more similar to Ca than it is to Ba, whereas the Brazil nut tree seems to have a unique capacity for accumulating Sr, Ba, and Ra in a similar way [39]. Generally, this pairing in chemical behavior can be easily explained by the ionic radii of the metal ions. Ca^2+^ (0.99 Å) and Sr^2+^ (1.12 Å) are very similar to each other, as are Ba^2+^ (1.34 Å) and Ra^2+^ (1.37 Å). However, the speciation of Sr in Brazil nuts has not yet been investigated. While Moreda-Pineiro et al. [20] determined a comparably low bioavailability for Ba, with 7.8% the bioavailability of Sr is much lower than we observed, likely for the same reason as mentioned above, namely that they used the significantly different technique of dialyzability.

Considering a maximum Sr content of approximately 200 µg/g (Table 1) and a bioaccessibility of 52% as determined in our study, daily consumption of one Brazil nut (5 g) would result in an intake of about 500 µg/day. There is no evidence of toxic effects of Sr in adults [2]. Furthermore, this value is well below the reference dose (RfD) of 0.6 mg/kgBW/day established by the U.S. EPA [32] and the minimal risk level (MRL) of 2.0 mg/kgBW/day set by the ATSDR [33], and therefore does not pose a health concern.

Moreover, the excretion pattern of Sr differs markedly from that of Ba and Ra, but resembles that of Ca. Sr is eliminated primarily via urine, with fecal excretion playing a smaller role (urine-to-feces ratio ~4 for Sr and ~2 for Ca). After 8 days, the cumulative excretion amounts to approximately 60% for Sr and about 40% for Ca [81,82]. These findings support the assumption that Sr exhibits chemical behavior more closely aligned with Ca than with Ba or Ra.

The lanthanides La and Eu are with 27% moderately bioaccessible. Also, for this group of metals (lanthanides) up to now, no speciation or binding form in Brazil nuts is known. Considering again a daily 5 g portion of one Brazil nut, about 0.7 µg/g Eu+La (Table 1) and 27% bioaccessibility, it would result in an intake of about 1 µg/day, far below the recommended upper intake of 70 µg/kgBW/day for REEs [44,45].

In vitro bioaccessibility methods provide a faster and more cost-effective alternative to in vivo bioavailability studies, yet the UBM approach applied here remains limited by its reliance on artificial digestive fluids. While the inclusion of microbiota or artificial membranes could improve approximation to true bioavailability, validation through empirical in vitro–in vivo correlations remains essential [55,56,83]. Another limitation of this study is the use of defatted Brazil nuts (BNF) instead of whole nuts, which represent the form typically consumed. Although BNF lacks certain matrix components that may additionally influence bioaccessibility, it provided a homogeneous substrate and yielded comparable results to whole nuts in selected experiments (e.g., Ba solubility). Despite these limitations, this study presents the first bioaccessibility data for several toxic metals in Brazil nuts, specifically Eu, La, and Ra, thereby offering an initial basis for evaluating their behavior and potential health risks following oral exposure.

### 2.3. Speciation of Se and Determination of Selected Organic Species by NMR

**Figure 3:**
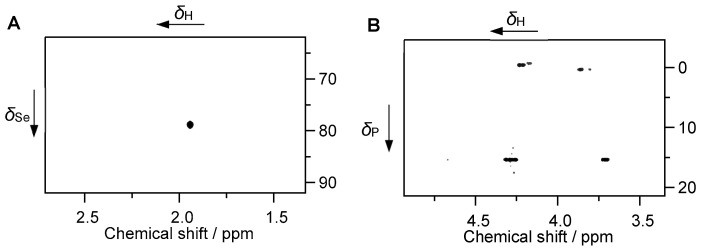
^1^H,^77^Se-HMBC NMR spectrum (SF0) showing the correlation signal associated with selenomethionine (**A**). ^1^H,^31^P-HMBC NMR spectrum (SF1) showing correlation signals associated with phosphate species (*δ*_P_ ~ 0 ppm) and phosphonate species (*δ*_P_ ~ 15 ppm) (**B**).

In order to complement the elemental analyses and assumptions reported above, we performed some NMR experiments on the BNF fraction soluble in diluted aqueous HCl, supplementing data on organic constituents. For doing so, at first the complete soluble fraction (denoted SF0) was used for ^77^Se NMR. In order to simplify the spectra and to separate constituents at least to some extent by polarity and molar weight using reversed-phase High Performance Liquid Chromatography (HPLC), SF0 was separated into four fractions (denoted SF1 through SF4) that were individually subjected to NMR (for details, see Section 3). From these fractions, as expected from the eluent composition by gradient, SF1 contains low molecular weight and/or highly polar substances, while SF2 is characterized by less polar substances, and SF3+SF4 contain high-molecular-weight substances like proteins, as corroborated by NMR spectral characteristics. Spectra with assignments of selected compounds are provided in Figure 3 and in Figures S2–S16, Supplementary Materials, together with references supporting the interpretation of NMR data [84,85,86,87,88,89,90,91,92,93]. Of course, the Brazil nut matrix is very complex and analyzing all its constituents is not only challenging but also far beyond the aim of this study. We thereby focused on the soluble fraction (discarding the insoluble remainder) as this portion is closer to the fluids associated with the digestive system. Nevertheless, we were able to assign some components of interest, which will be addressed in the following.

To ensure detectability of the presumably weak ^77^Se NMR signal (natural abundance of the NMR-active isotope is merely 7.6%), we measured the complete soluble fraction (SF0) and exploited polarization transfer from nearby (within few bonds) ^1^H nuclei in a heteronuclear multiple-bond correlation (HMBC) experiment. The NMR spectrum in Figure 3A discloses the correlation between methyl protons and Se, indicative of SeMet. Corresponding ^1^H and ^77^Se NMR chemical shifts, 1.94 and 78.8 ppm, respectively, are in excellent agreement with reported values, taking into account that the resonances of both ^1^H and ^77^Se are pH-dependent for the free amino acid as well as variant depending on the protein’s sequence and 3D structure [94,95,96]. Given the fact that the whole soluble fraction (SF0) obtained from extraction using aqueous HCl was containing remarkable amounts of (large) proteins, it is likely that SeMet was present as protein-bound amino acid. Obviously, the high Se contents in the Brazil nut powder are somehow connected to this amino acid. Our ^77^Se NMR experiments confirm the results recently obtained with X-ray absorption near-edge structure (XANES) [11] and HPLC [17,63], which also identified SeMet as the most abundant Se species in Brazil nuts.

^31^P NMR spectra also revealed notable amounts of phosphorus being present, indicating that the water-soluble P-containing compounds are either highly polar and/or small molecules (SF1) or associated with macromolecules such as proteins (SF3+SF4); see Figures S2 and S3, Supplementary Materials, for comparison. The lack of corresponding ^31^P signals in SF2 (less polar substances) arise from the small amount of substance. As depicted in Figure 3B, SF1 (highly polar and/or small molecules) reveals phosphate groups and one distinct phosphonate site resonating around *δ*_P_ ~ 0 ppm and at *δ*_P_ 15.4 ppm, respectively. ^31^P HMBC correlations to nearby ^1^H nuclei associated with phosphate monoesters O_3_P–O–CH_2_ characteristics are, in accordance with literature [97,98], assigned to phytate. As inferred from the chemical shift and phase of the corresponding ^1^H–^13^C heteronuclear single-quantum coherence (HSQC) NMR correlation signals, the phosphonate derivative features a O_3_P–CH(OH) moiety, similar to compounds reported in the literature [99]. Presence of a phosphonate is noteworthy since this class of substances is repeatedly observed in natural materials and in vivo, however its role or metabolism remains largely unanswered [100]. The phosphate monoester sites detected in SF3 and SF4 (macromolecules) and suspected to be present but not detected in SF2 (less polar substances), i.e., those fractions bearing high-molecular-weight molecules are ascribed to phytate strongly associated with peptides/proteins. Phytate is known to form complexes with proteins. At sufficiently low pH values (below the isoelectric point of the protein), the phytate anion binds strongly to the proteins’ cationic groups, i.e., the imidazole group of histidine, the guanidyl group of arginine, the ε-NH_3_^+^ group of lysine (see below), and the N-terminal α-NH_3_^+^ group have been implicated as binding sites [101].

In general, the increase in the average molecular weight along the fractions SF1 through SF4 can be seen from the shrinking resolution of the ^1^H NMR signals; see Figure S2A–D, Supplementary Materials. Upon increasing the molecular weight, not only the total number of ^1^H signals, but also the correlation time increases. The latter translates into significant shortening of the transversal relaxation time (*T*_2_), especially for ^1^H nuclei resulting in successive line broadening.

SF1, the fraction of small and/or polar molecules reveals well-known yet interesting components. That is, for instance, the hydroxycarboxylates citrate and lactate, the disaccharide sucrose, and the non-proteinogenic α-amino acid ornithine. We also detected a deoxy sugar acid, tentatively assigned to 2-deoxyribonic acid. The latter is speculated to be a degradation product of 2-deoxyribose, the pentose sugar contained in DNA, forming upon oxidizing the aldehyde group to a carboxyl group while the sugar is in open-chain form present during interconversion of anomers. Associated 2D NMR spectra are provided as Figures S4–S10, Supplementary Materials.

SF2 comprises a mixture of numerous compounds associated with reduced polarity. This fraction is rich in the amino acid tryptophan as easily recognized by its unique correlation pattern in the total correlation spectrum (TOCSY), comprising the six signals of the indole residue (two doublets and two triplets as well as two singlets associated with the six- and five-membered rings, respectively) as well as the corresponding H-α signal and the two H-β signals (see Figures S11 and S12, Supplementary Materials). Additionally, SF2 exhibits notable contents of the (peptide-bound) amino acids lysine and/or arginine, accompanied with a peculiar chemical shift of the resonance associated with the side chain terminal NH observed in SF2 TOCSY and SF4 ^1^H,^15^N-HSQC spectra depicted in Figures S13–S16, Supplementary Materials, respectively, ascribed to the interaction with phytate. From the moderate number of (yet strongly overlapping and thus poorly resolved) resonances in the NH/H-α spectral region (8.8–7.8 vs. 4.6–3.6 ppm), peptide chains in SF2 are shorter than those in SF4. The TOCSY of SF2 as well as both the TOCSY and ^1^H,^13^C-HSQC of SF4 display in their respective high-field regions manifold signals characteristic for aliphatic side-chain methyl group terminating amino acids such as valine, leucine, and isoleucine as well as small amounts of a water-soluble fraction with spin correlation patterns and chemical shifts characteristic of sterol derivatives.

In summary, ^77^Se NMR confirmed, for the first time in Brazil nuts, the occurrence of Se predominantly bound to the amino acid methionine as SeMet. ^31^P NMR verified the presence of phytate, which is assumed to complex alkaline earth metals such as Ba and Ra, potentially explaining their high solubility but low bioaccessibility. However, this interaction could not be conclusively demonstrated by this method, and the speciation of these metal ions in Brazil nuts remains unresolved. Furthermore, the water-soluble fraction contained a broad spectrum of compounds, ranging from small molecules to macromolecules with varying polarity. Most of these identified phytochemicals are known as bioactive compounds that help to reduce the risk of certain types of cancer or cardiovascular diseases and enhance immune function, especially in combination with the uniquely high Se content in Brazil nuts [3,5].

### 2.4. Speciation Determination of Eu in GIT by Luminescence Spectroscopy

The following important questions are addressed in more detail in this chapter: (i) whether and how the speciation of metal ions in the digestive system changes in the presence of food, and (ii) how the complexing ability of decorporation agents (DAs) used to promote the excretion of chemo- or radiotoxic metal ions is affected by food. These questions will be addressed experimentally using Eu as an example, as it is chemically similar to some highly radio- and chemotoxic trivalent actinides, An(III), such as americium (Am) or curium (Cm). Due to its unique luminescence properties, Eu(III) is often used as a non-radioactive model substance for these An. Therefore, the digestion procedure was repeated with BNF, and traces of Eu (10 µM) were added to all biofluids, as the naturally occurring concentration of Eu in Brazil nuts (see Table 1) was too low to yield useful speciation data by TRLFS. Defatted Brazil nut flour (BNF) was exclusively used for TRLFS measurements to provide a uniform and homogenized material, thereby ensuring comparability of results and reducing the higher matrix complexity associated with whole Brazil nuts.

The speciation of Eu in artificial biofluids of the digestive system has already been determined [58]. The Eu species were identified based on the UBM protocol with slight modifications using TRLFS in every single biofluid (saliva, gastric juice, pancreatic juice, and bile fluid) as well as in the mixtures within the stomach (saliva + gastric juice), small intestine (pancreatic juice + bile fluid) and in the whole digestion mixture of all four biofluids (GIT). In GIT, Eu is predominantly coordinated by the protein mucin and by phosphate and carbonate species [58].

**Figure 4:**
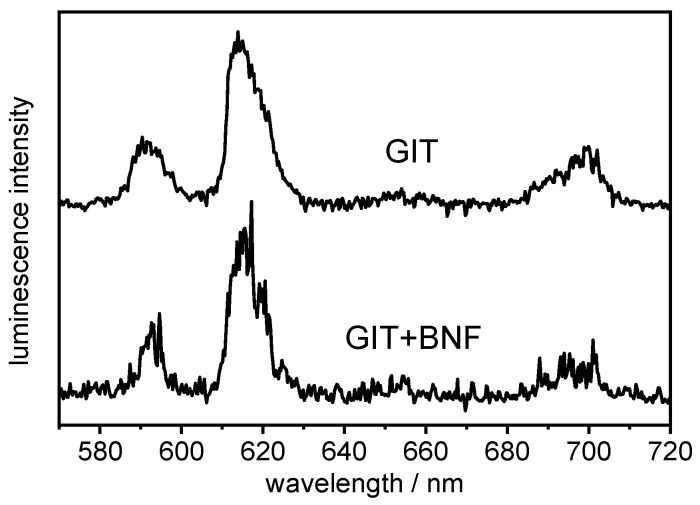
Luminescence spectra of 10 µM Eu in the artificial biofluids of the gastrointestinal tract (GIT) without and with Brazil nut flour (BNF) after simulated digestion.

Figure 4 shows the luminescence spectra of Eu in GIT without and with BNF, and important luminescence data are summarized in Table 3. In principle, the shape of the spectra is very similar, but the soluble substances of BNF effect a strong decrease in luminescence intensity. This might be due to quenching effects of either bioligands, which are involved in the Eu complexation, or iron, which is known to quench the Eu luminescence and is present in Brazil nuts in significant amounts of approximately 20–80 µg/g [2,9,13,16,18,19,21,24,37].

The luminescence lifetime of Eu in GIT is comparable to the literature values [58,102] (see Table 3; for lifetime spectra, see Figure S17, Supplementary Materials). It increases slightly from 235 ± 26 µs in the GIT mixture to 364 ± 16 µs in presence of BNF. This corresponds to four and two remaining water molecules, respectively, in the 9-fold spherical coordination sphere of Eu. This might be a hint that substances released from BNF (see NMR results, Section 2.3) additionally coordinate the Eu ion. However, due to the large number of soluble inorganic and organic substances released from BNF during the digestion procedure, it is not possible to specify the binding partner(s) more precisely within the scope of this study.

**Figure 5:**
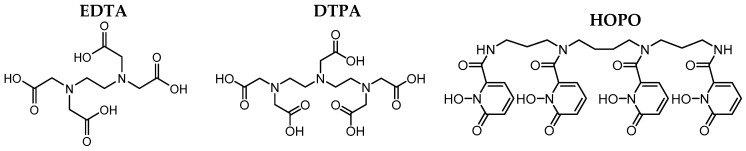
Structures of the investigated chelating agents ethylene diamine tetraacetic acid (EDTA), diethylene triamine pentaacetic acid (DTPA), and 3,4,3-LI(1,2-HOPO) (HOPO).

The impact of BNF on DAs in the GIT was investigated with the aminocarboxylates EDTA and DTPA and the spermine-based hydroxypyridinone HOPO (Figure 5). These chelating agents are, on the one hand, commercially in use for decorporation already for a long time (EDTA, DTPA), while HOPO is, on the other hand, a new promising chelator being currently in clinical trials [68,69,70]. Their complexing behavior towards Eu in the artificial biofluids of GIT have already been investigated [102]. It was shown that HOPO is a very strong complexing agent for Eu, comparable to DTPA and way stronger than EDTA, which renders it a highly potent DA.

**Figure 6:**
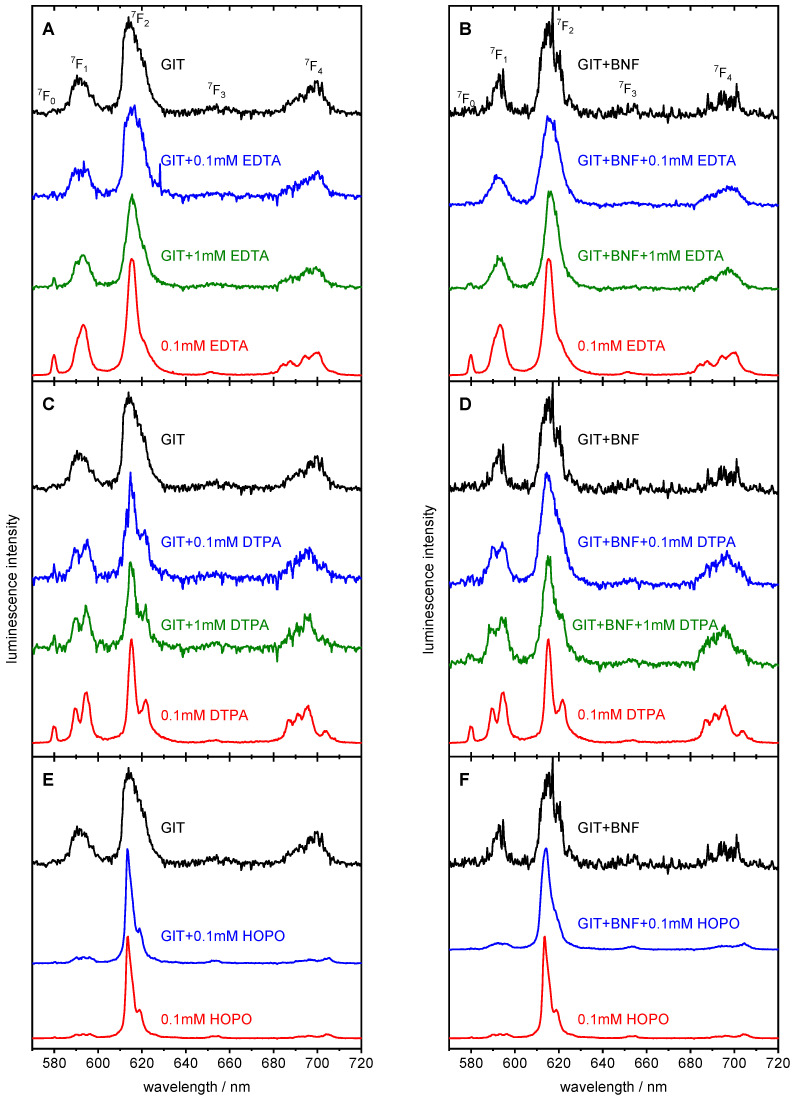
Luminescence spectra of 10 µM Eu^3+^ in GIT without (**A**,**C**,**E**) and with (**B**,**D**,**F**) Brazil nut flour (BNF) and with the chelating agents EDTA (**A**,**B**); DTPA (**C**,**D**), and HOPO (**E**,**F**) after digestion simulation at 37 °C (TRLFS measurements were carried out at RT).

Figure 6A–F show selected Eu luminescence spectra in GIT without (A, C, E) and with BNF (B, D, F) and the respective DA and, for comparison, the luminescence spectra of the corresponding Eu–DA complexes in aqueous solution. The characteristic luminescence data are listed in Table 3, and the corresponding luminescence decay curves are depicted in the Figures S18–S20, Supplementary Materials. The spectral features and luminescence lifetimes of the pure Eu complexes with EDTA, DTPA, and HOPO are in accordance with the literature data (Table 3).

The most important features of the Eu–EDTA luminescence spectrum are a pronounced ^7^F_0_ transition, a small shoulder on the right side of the ^7^F_2_ transition, and a 4-fold (2 + 2) splitting of the ^7^F_4_ transition (Figure 6A,B, red spectra). The luminescence lifetime corresponds to three water molecules remaining in the Eu surrounding, which points to the 6-fold coordination by EDTA. The EDTA-containing Eu spectra in GIT (Figure 6A) and GIT + BNF (Figure 6B) do not show these spectral features. Therefore, it can be qualitatively concluded that EDTA was not able to completely displace the ligands of GIT from Eu, even at its highest concentration of 1 mM. The displacing efficacy seems to be even lower in presence of BNF. This can be explained by a large variety of competitive anions and cations, especially Ca^2+^ in GIT, which is enlarged by the soluble substances from BNF. The luminescence lifetime of Eu is slightly enhanced by adding EDTA in both cases, GIT and GIT + BNF (see Table 3), indicating that EDTA is at least partly involved in the Eu coordination sphere. The Eu–DTPA luminescence spectrum shows, besides the pronounced ^7^F_0_ transition, a prominent 2-fold splitting of the ^7^F_1_ and ^7^F_2_ transitions as well as a 4-fold (3 + 1) splitting of the ^7^F_4_ transition (Figure 6C,D, red spectra). The luminescence lifetime is related to one remaining water molecule and an 8-fold coordination of the Eu ion by DTPA. Comparing the luminescence spectra in GIT (Figure 6C), the characteristic spectral features of the Eu–DTPA complex appears already at the lower DTPA concentration (0.1 mM, blue) and nearly fit them at the highest DTPA concentration (1 mM, green). The same effect can be observed for GIT + BNF (Figure 6D); however, the spectral shape associated with the pure Eu–DTPA complex is not matched. It can thus be concluded that substances released from BNF still contribute to the Eu coordination, and DTPA is not able to completely displace all competing bioligands. In all mixtures with DTPA, the luminescence lifetimes are shorter than that of the pure Eu–DTPA complex (see Table 3). This may be due to quenching by bioligands involved in Eu complexation or by iron, which is known to quench Eu luminescence and is present in Brazil nuts at 20–80 µg/g [2,9,13,16,18,19,21,24,37].

The luminescence spectrum of Eu–HOPO is characterized by an extremely intense ^7^F_2_ peak (Figure 6E,F, red spectra), which causes a very high ^7^F_2_/^7^F_1_ ratio (see Table 3). This spectral signature is dominant in GIT (Figure 6E) as well as in GIT + BNF (Figure 6F), even at the lowest HOPO concentration (0.1 mM, blue). The same luminescence lifetime, indicating one (or no) remaining water molecule and 8-fold HOPO coordination of Eu, appears in both the GIT and GIT + BNF mixture (see Table 3). One can conclude that in GIT, already at 0.1 mM HOPO concentration, the Eu was completely chelated. BNF decreases this effect only marginally. Compared to EDTA and DTPA, HOPO shows the best competition properties.

To summarize, these results qualitatively demonstrated that in the artificial biofluids of the digestive system, the chelating properties of the strong complexing agents EDTA, DTPA, and HOPO are retained in principle. Foodstuff like Brazil nuts showed only marginal influence on the complexation behavior. However, to quantify the effects, more comprehensive experiments, for example, including deuterated ligands as performed by Friedrich et al. [102,106], are necessary.

## 3. Materials and Methods

### 3.1. Chemicals and Materials

Brazil nuts and Brazil nut flour (BNF) were commercially obtained from an online trader (Brazil nut: 1 kg lot of whole nuts from Foodino, Bremen, Germany; origin: Amazon Region, country not specified; Brazil nut flour: 750 g lot from L-Carb-Shop, Köln, Germany; country of origin: Peru). The whole Brazil nuts were ground in a kitchen machine to a particle size of about 0.5–5 mm in our lab. BNF was used as purchased, which was produced from de-oiled nuts and grounded to a mesh size of <50 (≅297 µm) as stated on the packaging. For the bioaccessibility and speciation experiments, only defatted Brazil nut flour (BNF) was used, as it provides a uniform and homogenized material, thereby ensuring result comparability and reducing the greater matrix complexity associated with whole Brazil nuts. The following chemicals were used as obtained: H_4_EDTA (>99%), NaCl (99.5%), MgCl_2_∙6H_2_O (>99%), CaCl_2_∙2H_2_O (99%), KH_2_PO_4_ (≥99%), NaHCO_3_ (≥99%), glucose (p.a.), Na_2_SO_4_ (≥99%, all Roth, Karlsruhe, Germany); HNO_3_ (65%), H_2_O_2_ (30%), HCl (32%), KCl (p.a.), NaH_2_PO_4_∙H_2_O (anhydrous), KHCO_3_ (p.a.), D-(+)-glucosamine hydrochloride (99.5%, all Merck, Darmstadt, Germany); urea (99.5%), uric acid (99%, both Acros, Geel Belgium); EuCl_3_∙6H_2_O (99.99%), NH_4_Cl (99.5%, both Sigma-Aldrich, Taufkirchen, Germany); glucuronic acid (99.5%, Thermo Fisher, Dreieich, Germany); KSCN (p.a., Riedel-de Haen, Seelze, Germany); H_5_DTPA (>99%, Fluka-Feinchemikalien GmbH, Neu-Ulm, Germany); D_2_O (99.9% D, Deutero, Kastellaun, Germany). 3,4,3-LI(1,2-HOPO) was synthesized as previously reported [102] and kindly provided by the group of Prof. Clemens Walther from Leibniz University of Hannover, Institute of Radioecology and Radiation Protection. Stock solutions were prepared by weighing and dissolving appropriate amounts of the respective chemical in Milli-Q H_2_O (18.2 MΩ cm, Millipore, Merck, Darmstadt, Germany). The enzymes α-amylase (porcine pancreas), pancreatin (porcine pancreas), trypsin (bovine pancreas), lipase (Rhizopus oryzae), and bile extract (bovine, all Sigma-Aldrich, Taufkirchen, Germany); mucin (porcine gastric mucosa, 75–95%, Roth, Karlsruhe, Germany); and pepsin (porcine stomach, Thermo Fisher, Dreieich, Germany) were weighted and added as obtained. The required pH values were adjusted with HCl (1.0, 0.1, and 0.01 M) and NaOH (1.0, 0.1, and 0.01 M) using a pH meter (inoLab pH 730, Xylem, Weilheim, Germany) equipped with a pH electrode (SCHOTT, BlueLine, SI Analytics, Mainz, Germany).

### 3.2. Simulation of Digestive Process

In Table 4, all components of the single artificial biofluids saliva, gastric juice, pancreatic juice, and bile fluid as well as the resulting mixture of the complete gastrointestinal tract (GIT) are listed.

The digestion process was simulated as depicted schematically in Figure 1. All four digestive fluids were preheated to 37 °C in a shaker before use. 10 mL preheated saliva was added to 0.5 mg BNF, and the mixture was shaken and incubated at 37 °C for 15 min. For the gastric phase simulation, 13.5 mL gastric fluid was incorporated into the saliva mixture, which was then incubated at 37 °C for 2 h while being shaken to mimic peristalsis. 27 mL pancreatic juice and 9 mL bile fluid were added and incubated for another 2 h to simulate the whole GIT. At the end of each digestive phase, 1 mL of the mixture was sampled for ICP-MC analysis. The samples were centrifuged for 10 min at 13,200 rpm, and the liquid phase was collected. Distilled HNO_3_ was then added to slightly acidify the samples for ICP-MS analysis. Each examination was performed in triplicate.

### 3.3. Inductively Coupled Plasma-Mass Spectrometry (ICP-MS)

Three defined mass aliquots of 0.30–0.37 g of ground Brazil nuts and three defined mass aliquots of 0.11–0.31 g of BNF, respectively, were digested in a laboratory microwave (ETHOS.lab, MWS Mikrowellensystem, Leutkirch, Germany) in a mixture of 4 mL 65% HNO_3_ and 1 mL 30% H_2_O_2_ at 210–215 °C. The resulting solutions were filled up to a volume of 25 mL with Milli-Q water for ICP-MS analysis.

For the Ba extraction experiments, mass aliquots of 5 g of the ground nuts and the flour, respectively, were stirred with 50 mL of 0.01 M HCl, 0.1 M HCl, or 1.0 M HCl for 6 h, 15 h, and 24 h at room temperature (20–22 °C). After the corresponding contact time, the liquid phase was separated via paper filter (Whatman, grade 597) and the filter cake was washed with a further 50 mL of HCl of corresponding concentration, resulting in a final volume of 100 mL. In case of a white precipitate after some standing time, the solution was furthermore separated via a cellulose-acetate filter with 0.45 µm pore width (LABSOLUTE, Th. Geyer GmbH & Co. KG, Renningen, Germany). Before ICP-MS measurement, aliquots were spiked with Rh as internal standard and diluted with 1.5–3% HNO_3_ to defined volumes.

Determination of Se, Sr, Ba, Eu, and La from microwave digestion and Ba from extraction experiments were carried out according to DIN EN ISO 17294-2:2017-01 [107] with a high-resolution sector field mass spectrometer with inductively coupled plasma (ELEMENT2, Thermo Fisher Scientific, Bremen, Germany) and with Ar as plasma gas. The extracted part was calculated by dividing the absolute mass determined from the measured concentration of Ba in the filtrate by the maximal leachable amount of Ba from a 5 g aliquot determined from the measurement of Ba in the microwave digestion solutions.

Samples from digestion process were slightly acidified with distilled HNO_3_ for ICP-MS analysis. Samples were determined with a quadrupole mass analyzer iCap RQ (Thermo Fisher Scientific, Bremen, Germany) with 1550 W RF-power, Ar as plasma gas, and Sc and Rh as internal standards. Each examination was performed in triplicate.

### 3.4. Gamma Spectrometry

In preparation of the gamma spectrometry, five mass aliquots of the ground whole Brazil nuts (48.3–50.7 g range) and one mass aliquot of BNF (71.1 g) were filled into a round plastic container with a diameter of 70 mm and a height of 21.5 mm. The containers were welded gastight (Rn) into a plastic bag. The gamma spectrometric measurements were carried out on n-type semiconductors of high purity germanium (HPGe) with a relative detection efficiency of 20–30% and measurement times between 70,000 and 103,000 s. Measurements were carried out according to DIN EN ISO 20042 VDE 0493-2042: 2022-06 [108] and the spectrum evaluations are executed with the software packages GammaVision (AMETEK/ORTEC, Oak Ridge, TN, USA) and InterWinner (ITECH Instruments, Vitrolle, France), respectively.

The radium isotope ^226^Ra can be directly determined via its emission line at 186.2 keV (3.6%) and indirectly via the ^222^Rn daughter nuclides ^214^Bi with the emission lines at about 609 keV (45.4%) and 1764 keV (15.3%) as well as ^214^Pb with the emission lines at 242 keV (7.3%), 295 keV (18.4%), and 352 keV (15.8%). The direct measurement of ^226^Ra can be disturbed by an interference with the emission line of ^235^U at 185.7 keV (57.2%), which is either corrected directly, by subtraction, or indirectly, via ^234^Th under assumption of natural U isotopic ratios. Otherwise, the line activities of ^226^Ra, ^214^Pb, and ^214^Bi should be in accordance with each other.

The radium isotope ^228^Ra is indirectly determined via its daughter nuclide ^228^Ac, with the emission lines at about 338 keV (11.3%), 911 keV (25.8%), and 969 keV (15.8%).

### 3.5. Alpha Spectrometry

For ^226^Ra analysis with alpha spectrometry after digestion, whole volumes of GIT mixture were used. After the digestive process simulation, the mixtures were centrifuged to prepare the analysis further. The experiment was triplicated.

A volume of 15 mL or 25 mL was taken from each sample, spiked with ^229^Th tracer and 5 mg Fe^3+^. After addition of about 15 mL Aqua Regia, the mixture was wet digested by heating on a hot plate at 175–200 °C for 4 h. After cooling, (NH_4_)_2_HPO_4_ solution and ammonia were added for coprecipitation of Fe(OH)_3_ and CaHPO_4_ at pH 8–9. The precipitate was ashed at 450 °C for 8 h. After dissolution in dilute HNO_3_, extraction chromatography via TEVA resin (TRISKEM, Rennes, France) was carried out to remove Th, while Ra was not retained by the resin. The eluate was evaporated to dryness, and the nitrate salts were transformed to their chloride form by adding 10–12 M HCl. The chloride residue was redissolved in a mixture of a diluted HCl/EDTA solution. With that solution, a cation exchange chromatography via DOWEX 50XWx8 was carried out to remove several matrix elements like Mg, Ca, Fe, Al, Mn, U, and Ac as well as traces of Th by several washing steps. Sr is only partially removed. Ra and Ba were not separated from each other in this step because of their chemical similarity. Ra together with Ba was eluted with 6 M HCl and 4 M HNO_3_. The Ra fraction was evaporated to dryness. After dissolution of the hardly observable residue in dilute HNO_3_, Ba was removed in a further extraction chromatography by retention on SR resin (TRISKEM, Rennes, France). The almost certainly Ba-free Ra fraction was evaporated to dryness with a small volume of (NH_4_)_2_C_2_O_2_ to bind Ra in the matrix and prevent it from adsorbing on the glass walls.

After dissolution in a mixture of 0.34 M (NH_4_)_2_C_2_O_2_ and 0.3 M HCl, Ra was electrodeposited on a stainless-steel plate at 0.6 A for 2 h in the presence of (NH_4_)_2_(PtCl_6_). The electrodeposition was stopped with 0.5 mL ammonia. The steel plate was demounted from the cell and washed with deionized water and acetone. After heating it for 10–15 min on a hot plate at 175–220 °C, the plate was positioned in the measurement chamber of an alpha spectrometer (AlphaAnalyst of Mirion (Canberra), Rüsselsheim, Germany).

There were two measurements necessary for the determination of the chemical yield as well as the ^226^Ra activity. In the first measurement immediately after electrodeposition, the plate is measured for 1 d to determine the initial activity of ^225^Ac from the virtual tracer nuclide ^225^Ra, the direct daughter nuclide of ^229^Th. In the second measurement 10–18 d after electrodeposition, the alpha emitting daughter nuclides ^225^Ac, ^221^Fr, ^217^At, and ^213^Po have increased to an easily measurable amount, whereby ^217^At is chosen for evaluation of the chemical yield, as it stands undisturbed from other alpha nuclides in the alpha spectrum. The second measurement is carried out for 7 d.

### 3.6. NMR Spectroscopy

Samples dedicated to NMR spectroscopic investigation were prepared by extracting 5 g of BNF with 15 mL of 0.1 M HCl for 7 d in an overhead shaker. The suspension was then centrifuged to separate the insoluble fraction, which was discarded. The clear supernatant was lyophilized, and the remaining pale-yellow powder was then re-dissolved in 700 µL D_2_O (SF0, pD = 5) and subjected to ^77^Se NMR measurement. Afterwards, the complete extract (SF0) was then separated into four distinct fractions (SF1–SF4) using automated column chromatography (Isolera Four, Biotage, Uppsala, Sweden) with reversed phase cartridges (Sfär C18 D, 12 g) and a solvent gradient consisting of acetonitrile/water + 0.1% trifluoroacetic acid (TFA) each (0% acetonitrile over 3 column volumes (CV), 0 → 15% acetonitrile over 25 CV, 100% acetonitrile over 7 CV with a flow rate of 12 mL/min). After lyophilization, the four fractions SF1 (164 mg), SF2 (3.7 mg), SF3 (9.5 mg), and SF4 (48.5 mg) were, respectively, dissolved in 90/10 (*v*/*v*) H_2_O/D_2_O (resulting pH ~ 2 owing to TFA contents) and individually investigated by different NMR techniques.

NMR spectra were obtained on an Agilent DD2-600 NMR system (Agilent Technologies, Waldbronn, Germany), operating at 14.1 T with corresponding ^1^H and ^13^C resonance frequencies of 599.8 and 150.8 MHz, respectively, using a 5 mm oneNMR^™^ probe, at (25 ± 0.2) °C.

^1^H NMR spectra were recorded using solvent signal suppression by pre-saturation for 2 s on the water resonance, followed by full spectral excitation applying a 2.7 µs (π/6) pulse, an acquisition time of 3 s, accumulating at least 32 scans using 3 s relaxation delay. ^31^P NMR spectra were obtained upon excitation by a 3.3 µs (π/6) pulse, accumulating 128–3000 scans depending on the sample amount of the individual fractions (SF0–SF4), applying inverse-gated ^1^H-decoupling during 1 s of FID acquisition, followed by 4 s relaxation delay. Heteronuclear single-quantum coherence (HSQC) and heteronuclear multiple-bond correlation (HMBC) sequences applied gradient-selection and adiabatic pulses. ^1^H,^13^C-HSQC and -HMBC spectra were acquired with 2048 × 1024 complex points in *F*_2_ and *F*_1_, 64 transitions per *F*_1_ increment, with a relaxation delay of 1 s, respectively, opting (2 × *J*)^−1^ polarization transfer delays of 3.45 and 62.5 ms, corresponding to 145 Hz ^1^*J* and 8 Hz ^n^*J*, respectively. ^1^H,^31^P- and ^1^H,^77^Se-HMBC spectra were acquired with 2048 × 128 and 2048 × 256 complex points in *F*_2_ and *F*_1_, 16 and 128 transitions per *F*_1_ increment, with a relaxation delay of 1 s, respectively, opting a (2 × *J*)^−1^ polarization transfer delay of 41.67 ms, corresponding to 12 Hz ^n^*J.*

For ^1^H–^1^H correlation, zero-quantum-filtered homonuclear total correlation spectra (TOCSY, mixing time 80 ms) were acquired using 2048 × 512 complex points in *F*_2_ and *F*_1_, 32 transitions per *F*_1_ increment and a relaxation delay of 1 s. Two-dimensional NMR experiments applied 1 s pre-saturation selective pulse on the water resonance for solvent suppression. Chemical shifts (*δ*) are reported in parts per million (ppm) relative to external TMSP-*d*_4_ (sodium 3-(trimethylsilyl)-2,2,3,3-tetradeuteropropionate) in D_2_O (*δ*_H_ and *δ*_C_ 0.0 ppm) as well as relative to external 85% H_3_PO_4_ (δP = 0.0 ppm).

### 3.7. Luminescence Spectroscopy

The biofluids were spiked with 10 µM Eu(III) before the digestion process simulation. Two preheated 4.5 mL saliva samples were prepared, and 0.25 g of BNF was added to one of the samples. Afterwards, 6.75 mL of gastric juice was added to both, completing the stomach mixture. Subsequently, 13.5 mL of pancreatic juice and 4.5 mL of bile juice were added to form the GIT mixture. The digestion process was simulated as described in Section 3.2. Before measurement, the samples were centrifuged for 10 min at 13,200 rpm, and only the liquid phase was used.

For the TRLFS measurements, a diode-pumped solid-state laser (NT230, Ekspla, Vilnius, Lithuania, 1.3 mJ/pulse) with a pulse duration of 5 ns and an excitation wavelength of 394 nm was used for excitation at room temperature. Emission spectra were detected with a spectrograph (SR-303i-A, Andor, Belfast, UK) and equipped with an ICCD camera (AndoriStar, DH320T-18U-63, Andor, Belfast, UK). The experimental setup included the following parameters: input slit width ranging from 50 to 300 μm, gate width of 0.5 ms, gain between 2500 and 3000, exposure time of 0.0123 s, and 200 accumulations. To capture time-resolved luminescence spectra, the delay between the laser pulse and camera control was sampled across 30 dynamic time intervals, spanning from 1 to 2000 μs.

The Eu(III) TRLFS spectra were analyzed using Origin 2019, version 9.6.0.172 (OriginLab Corporation, Northhampton, MA, USA). All Eu spectra were normalized to the area of the ^5^D_0_ → ^7^F_1_ transition (585–600 nm) to facilitate comparison to the ^5^D_0_ → ^7^F_2_ transitions.

The lifetimes of the luminescence species were determined by the following exponential decay function:

(1) $$E\left(t\right)=\sum_{i}E_{i}\times exp(-\frac{t}{\tau_{i}})$$

where *E* is the total luminescence intensity at the time *t*, *E*_i_ the luminescence intensity of the species i at *t* = 0, and *τ_i_* the corresponding luminescence lifetime.

With the obtained luminescence lifetimes, the number of water molecules in the first coordination sphere of Eu(III) can be estimated using the following equation [109,110] with luminescence lifetime *τ* in ms:

(2) $$n\left(H_{2}O\right)\pm 0.5=\frac{1.07}{\tau }-0.62$$

## 4. Conclusions

The bioaccessibility of Se, Sr, Ba, Ra, Eu, and La from Brazil nuts was assessed in vitro, based on the unified bioaccessibility method (UBM), developed by the Bioaccessibility Research Group of Europe (BARGE) [58,59,60]. This physiologically based extraction test, which incorporates a mixture of inorganic, organic, and enzymatic components (see Table 4), has been validated for the assessment of toxic metals and metalloids in soils [56,59,83,111] and, more recently, adapted for food matrices [112,113,114,115,116,117,118,119,120,121].

While bioaccessibility data for Se, Sr, and Ba already existed, this study provides the first in vitro bioaccessibility values for the radionuclide ^226^Ra as well as the REEs Eu and La in Brazil nuts.

Selenium showed a high bioaccessibility of approximately 85%, which can be attributed to its presence as the highly soluble and stable compound selenomethionine (SeMet), which was confirmed by multinuclear (^1^H, ^77^Se) NMR spectroscopy.

In contrast, Ba demonstrated a low bioaccessibility of about 2%, likely due to its binding to phytate, which, despite being highly soluble in HCl, results in limited intestinal availability. A similarly low bioaccessibility was observed for ^226^Ra. Eu and La exhibited a moderate bioaccessibility of around 27%, which may be representative for Ln(III) and An(III) known for their chemo- and radiotoxic potential.

Given the toxicological relevance of these elements, effective decorporation strategies are essential. Importantly, this study demonstrates that the speciation of Eu(III)—used here as a chemical analog for Ln(III) and An(III)—and the binding efficacy of selected chelating agents (EDTA, DTPA, and HOPO, see Figure 5) toward Eu(III) remains nearly unaffected by the food matrix.

When compared with the upper intake level recommendations provided by EFSA for Se [7], by EPA and ATSDR for Sr and Ba [30,31,32,33], as well as with the general maximum permissible dose limit for radioactivity for Ra [34], all individual maximum bioaccessibility values were found to be below these thresholds. This indicates that the daily consumption of a single Brazil nut is unlikely to pose a health risk, even over long-term intake. Nevertheless, despite the high excretion rates and relatively short biological half-lives of Ba and Ra, a residual toxicological risk persists due to their partial retention in bone tissue—particularly in the case of Ra, owing to its radioactivity.

Overall, this study contributes valuable insights into the bioaccessibility and potential health risks of trace elements in Brazil nuts, highlighting element-specific differences in gastrointestinal availability and their implications for dietary exposure assessment and risk evaluation.

## Figures and Tables

**Table 1:** Measured concentrations (*c*) of selected trace elements and heavy metals as well as specific activities (*a*) of radionuclides (RN) in defatted Brazil nut flour (BNF) and whole Brazil nuts and comparison with the literature values.

	*c* in Defatted BNF (µg/g)		*c* in Whole Brazil Nut (µg/g)	
**Element**	**Present Work**	**Literature**	**Present Work**	**Literature**
Se	10.1 ± 0.8	2–84 [13,15,18,22,23]	1.91 ± 0.25	1–55 [8,9,10,12,14,15,16,19,20,21,22,23,24,38]
Sr	760 ± 40	188–426 [18,22,23]	230 ± 13	115–198 [20,21,22,23,24,37]
Ba	8000 ± 400	2200 –7097 [18,22,23]	1720 ± 90	49–2476 [19,20,21,22,23,24,35,37]
La	0.122 ± 0.008		0.0291 ± 0.002	0.0014 [21]
Eu	2.49 ± 0.17		0.65 ± 0.03	<0.0004 [21]
**Radionuclide**	***a* (mBq/g)**			
^226^Ra	74.0 ± 5.3		51.6 ± 3.7	17–205 [12,25,38,39,40,41,42]
^228^Ra	63.0 ± 4.6		47.7 ± 3.0	18–100 [12,25,38,39,40,42]

**Table 2:** Concentrations (*c*) of selected trace elements and heavy metals, and specific activities (*a*) of radionuclides (RN) in defatted Brazil nut flour (BNF) before and after various digestion steps, including bioaccessibility (release) and comparison with literature values; p.w. = present work.

	Element Concentrations *c*/RN Activities *a*/Bioaccessibility (%)						
	BNF ^a^	Saliva		Stomach		GIT ^b^	
	*c* (µg/g)	*c* (µg/g)	Release (%)	*c* (µg/g)	Release (%)	*c* (µg/g)	Release (%)
**Se**	9.9 ± 0.9	3.5 ± 0.2	35 ± 2	5.7 ± 0.3	57 ± 3	8.4 ± 0.5	84 ± 5 (p.w.) 70 ± 23 [14] 74 [8] 19 ± 2 [20]
**Sr**	640 ± 30	143 ± 22	23 ± 3	337 ±16	53 ± 3	332 ± 29	52 ± 5 (p.w.) 7.8 ± 0.8 [20]
**Ba**	7100 ± 400	12 ± 2	0.18 ± 0.03	27 ± 6	0.38 ± 0.08	132 ± 5	1.9 ± 0.1 (p.w.) 2.2 ± 0.1 [20]
**La**	0.031 ± 0.003	0.003 ± 0.002	8 ± 6	0.005 ± 0.004	17 ± 13	0.008 ± 0.004	27 ± 13
**Eu**	0.47 ± 0.05	0.028 ± 0.001	6.0 ± 0.2	0.029 ± 0.005	6.2 ± 1.1	0.13 ± 0.03	27 ± 6
	***a* (mBq/g)**					***a* (mBq/g)**	
**^226^Ra**	74 ± 5					1.4 ± 0.3	1.9 ± 0.4

^a^ values of the BNF sample used for digestion; ^b^ digestion mixture of all four biofluids referred to as gastrointestinal tract fluid, GIT.

**Table 3:** Luminescence data of Eu(III) in artificial digestive system in absence and presence of BNF and the chelators EDTA, DTPA, and HOPO (10 µM Eu, pH = 6.5 ± 0.5, RT) and comparison with data from the literature.

Sample (+10 µM Eu)	pH	Lifetime (µs)	n (H_2_O) ± 0.5	^7^F_2_/^7^F_1_ Intensity Ratio	Reference
GIT	6.84	235 ± 26	4.0	3.4	p.w. ^a^
	6.8	261 ± 11 // 1300 ±32			[58]
	6.5	315 ± 52			[102]
GIT + BNF	6.66	364 ± 16	2.3	3.8	p.w.
GIT + 0.1 mM EDTA	6.49	250 ± 23	3.7	3.4	p.w.
GIT + 1 mM EDTA	6.54	314 ± 12	2.8	3.0	p.w.
GIT + BNF + 0.1 mM EDTA	6.51	419 ± 30	1.9	3.2	p.w.
GIT + BNF + 1 mM EDTA	6.48	557 ± 27	1.3	3.3	p.w.
0.1 mM EDTA	6.44	317 ± 3	2.8	2.4	p.w.
	6.5	326 ± 8			[102]
	3–9	299 ± 6			[103]
	4–6	307			[104]
GIT + 0.1 mM DTPA	6.58	377 ± 31	2.2	2.7	p.w.
GIT + 1 mM DTPA	6.52	372 ± 36	2.3	2.1	p.w.
GIT + BNF + 0.1 mM DTPA	6.54	389 ± 22	2.1	2.9	p.w.
GIT + BNF + 1 mM DTPA	6.54	422 ± 31	1.9	2.1	p.w.
DTPA	6.53	628 ± 19	1.1	1.9	p.w.
	7.4	618 ± 4	1.2	1.9	[52]
	6.5	545 ± 81			[102]
	2–5	577			[104]
GIT + 0.1 mM HOPO	6.48	689 ± 24	1.0	10.2	p.w.
GIT + BNF + 0.1 mM HOPO	6.51	527 ± 16	1.4	10.5	p.w.
HOPO	6.47	661 ± 27	1.0	12.2	p.w.
	7.4	829 ± 4	0.8	11.7	[52]
	6.5	713 ± 58			[102]
	7.4	805 ± 81			[105]

^a^ present work.

**Table 4:** Compositions of single biofluids as well as the resulting mixture of the gastrointestinal tract (GIT) following the slightly modified UBM protocol [58,59].

Components	Saliva	Gastric Juice	Pancreatic Juice	Bile Fluid	GIT
**Inorganics (mmol/L)**					
NaCl	10.2	94.2	234	180	159
KCl	24.0	22.1	15.1	10.1	17.3
NH_4_Cl	-	11.4	-	-	2.63
MgCl_2_	-	-	0.5	-	0.23
CaCl_2_	1.0	-	1.4	1.5	2.28
NaH_2_PO_4_	14.8	3.9	-	-	3.18
KH_2_PO_4_	-	-	1.2	-	0.55
NaHCO_3_	-	-	133.5	137.7	82.8
KHCO_3_	15.0	-	-	-	2.31
Na_2_SO_4_	8.0	-	-	-	1.23
KSCN	4.1	-	-	-	0.63
**Organics (mmol/L)**					
urea	6.7	2.8	3.3	8.3	4.58
uric acid	0.1	-	-	-	0.02
glucose	-	7.2	-	-	1.66
glucosamine∙HCl	-	3.1	-	-	0.72
glucuronic acid	-	0.2	-	-	0.05
**Enzymes (mg/mL)**					
α-amylase	1.0	-	-	-	0.15
mucin	0.5	3.0	3.0	-	2.15
pepsin	-	1.0	-	-	0.23
pancreatin	-	-	3.0	-	1.85
trypsin	-	-	1.0	-	0.46
lipase	-	-	0.5	-	0.23
bile extract	-	-	-	6.0	0.92
pH	6.5 ± 0.5	1.0 ± 0.2	7.4 ± 0.2	8.0 ± 0.2	6.5 ± 0.5

## Data Availability

The original contributions presented in this study are included in the article/Supplementary Material. Further inquiries can be directed to the corresponding author. The raw data supporting the conclusions of this article will be made available by the authors on request.

## Supplementary Materials

The following supporting information can be downloaded at https://www.mdpi.com/article/10.3390/ijms26178312/s1.

## Abbreviations

The following abbreviations are used in this manuscript:

An	Actinides
ATSDR	Agency for Toxic Substances and Disease Registry
BARGE	Bioaccessibility Research Group of Europe
BNF	Brazil nut flour
BW	Body weight
DA(s)	Decorporation agent(s)
DTPA	Diethylenetriaminepentaacetic acid
EDTA	Ethylenediaminetetraacetic acid
EFSA	European Food Safety Authority
EPA	U.S. Environmental Protection Agency
GIT	Gastrointestinal tract
HMBC	Heteronuclear multiple-bond correlation
HOPO	1,5,10,14-tetra(1-hydroxy-2-pyridon-6-oyl)-1,5,10,14-tetraazatetradecan3,4,3-LI(1,2-HOPO)
HPLC	High Performance Liquid Chromatography
HSQC	Heteronuclear single-quantum coherence
ICP-MS	Inductively coupled plasma-mass spectrometry
Ln	Lanthanides
NMR	Nuclear magnetic resonance
REE(s)	Rare earth element(s)
RN	Radionuclide
SF(0…4)	Soluble fraction(0…4)
TOCSY	Total Correlation Spectroscopy
TRLFS	Time-resolved laser-induced fluorescence spectroscopy
UBM	Unified bioaccessibility method
WHO	World Health Organization
