# Supplementary material for: In Vitro Bioaccessibility and Speciation of Toxic and Nutritional Trace Elements in Brazil Nuts
Source: Int J Mol Sci. 2025 Aug 27;26(17):8312. doi: 10.3390/ijms26178312 (PMC12428530; doi:10.3390/ijms26178312)
Supplement: Supplementary file 1 [file ijms-26-08312-s001.zip › ijms-3816930-supplementary.pdf]

SUPPLEMENTARY MATERIAL

In vitro bioaccessibility and speciation of toxic and nutritional trace elements in Brazil nuts

Astrid Barkleit*[1], Jiyoung Eum[1], Diana Walther[2], Daniel Butscher[1], Sebastian Friedrich[1], Katharina Müller[1], Jerome Kretzschmar[1]

[1] Helmholtz-Zentrum Dresden–Rossendorf, Institute of Resource Ecology, 01328 Dresden, Germany

[2] VKTA - Radiation Protection, Analytics & Disposal Rossendorf Inc., 01328 Dresden, Germany

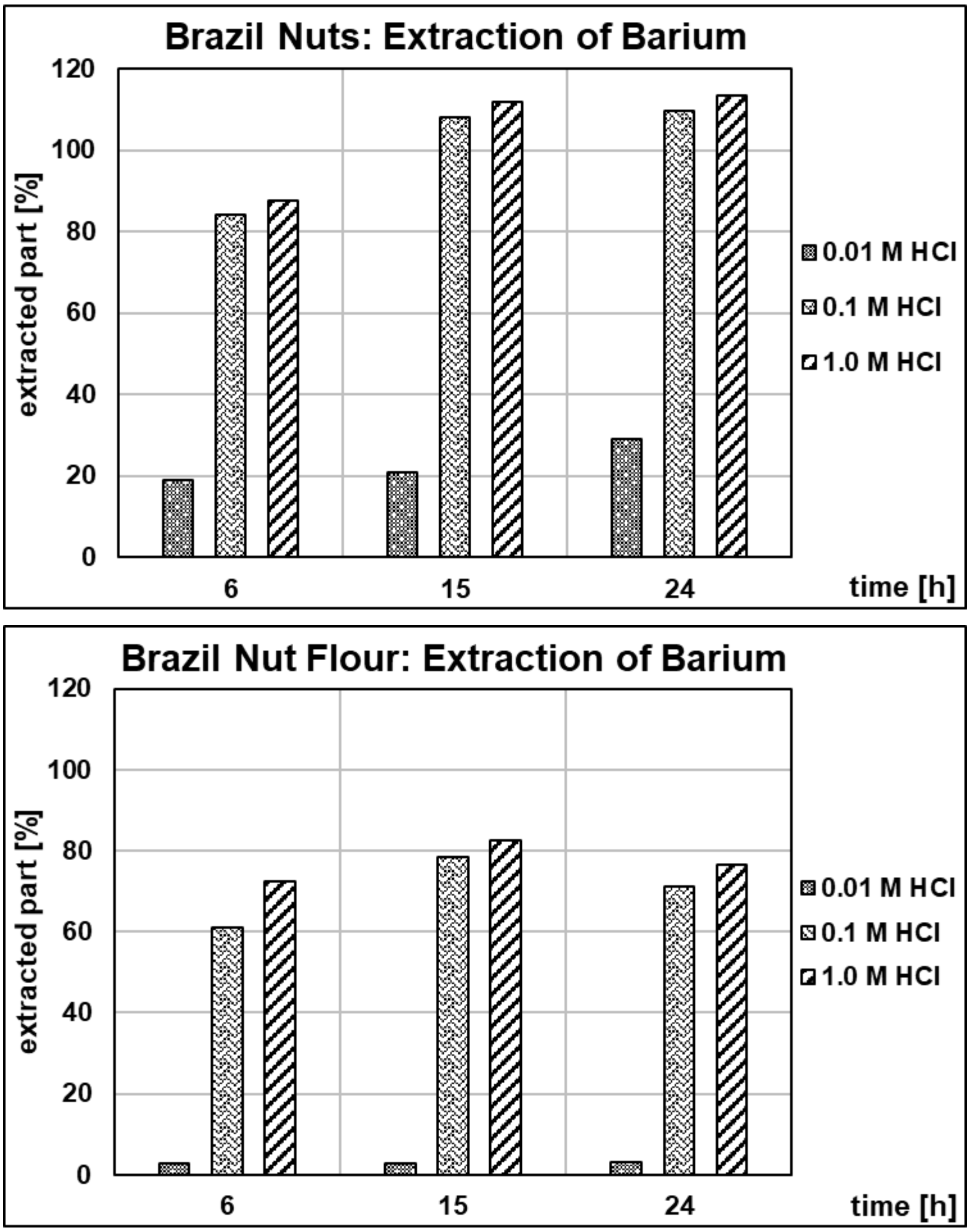

Figure S1. Extraction of barium from Brazil nuts and Brazil nut flour as a function of HCl concentration and extraction duration.

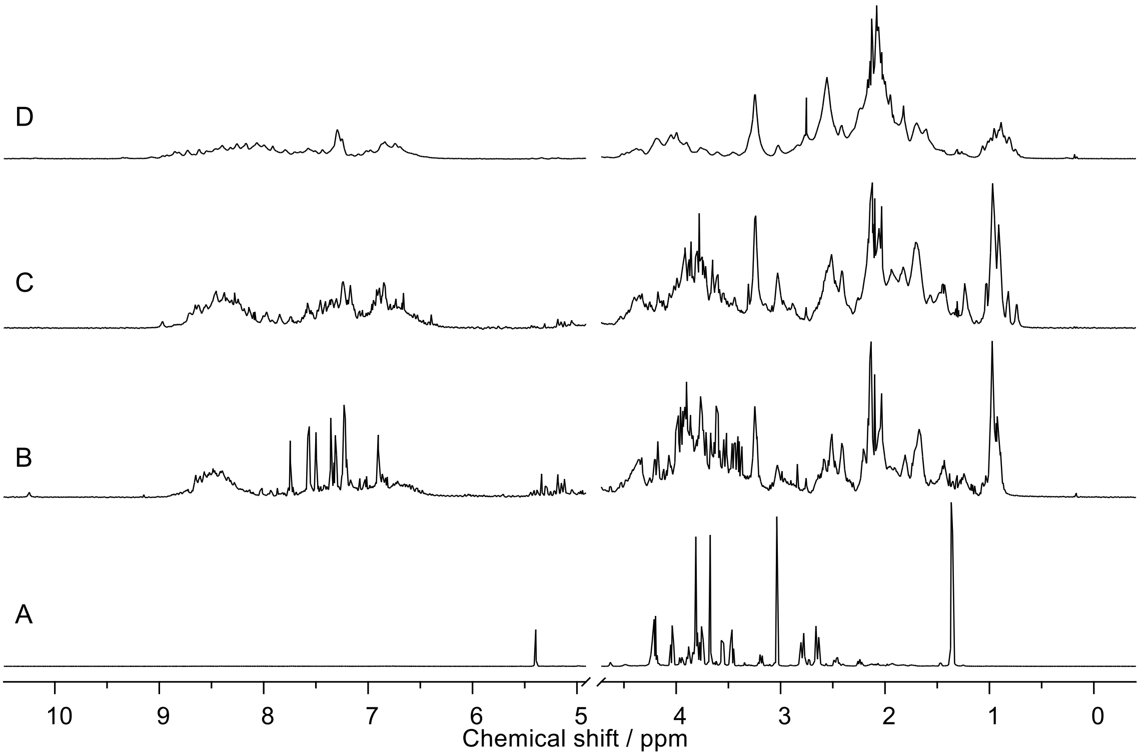

Figure S2. ^{1}H NMR spectra of the fractions obtained from HPLC: SF1 (164 mg, A), SF2 (3.7 mg, B), SF3 (9.5 mg, C), and SF4 (48.5 mg, D) respectively dissolved in 90/10 H_2O/D_2O. The water signal was suppressed by a pre-saturation sequence and, for clarity, cut in the displayed spectra.

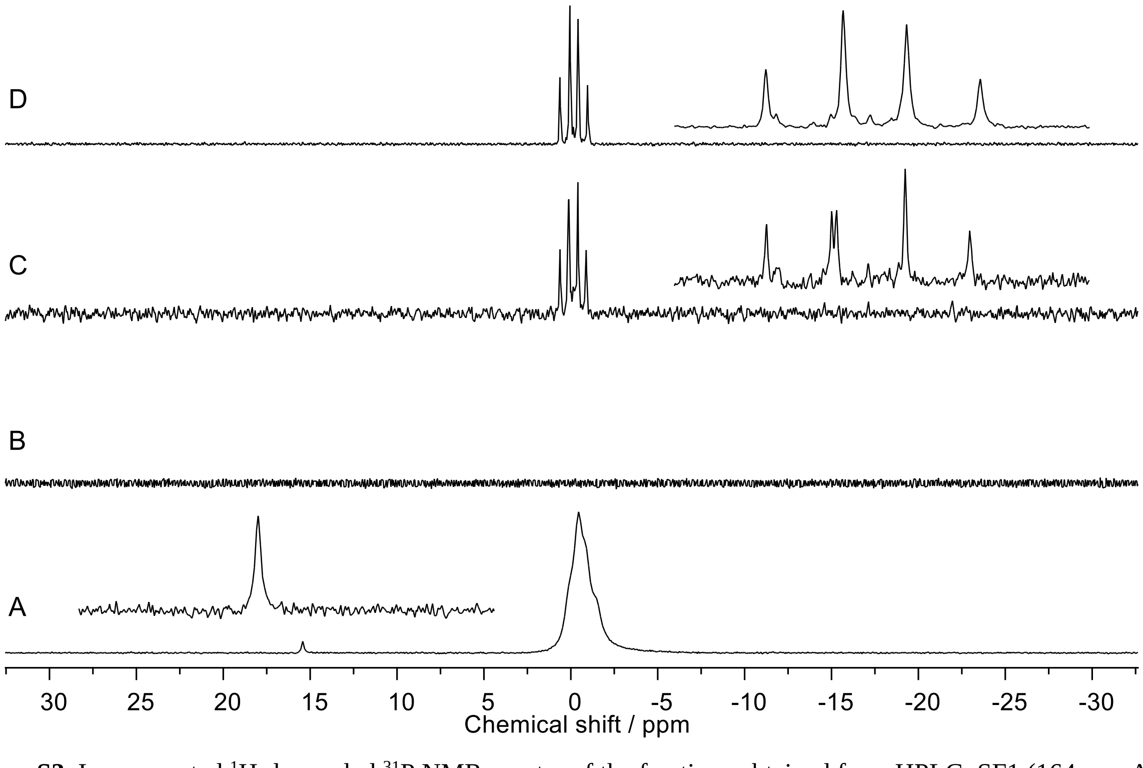

Figure S3. Inverse-gated ^{1}H-decoupled ^{31}P NMR spectra of the fractions obtained from HPLC: SF1 (164 mg, A), SF2 (3.7 mg, B), SF3 (9.5 mg, C), and SF4 (48.5 mg, D) respectively dissolved in 90/10 H_2O/D_2O.

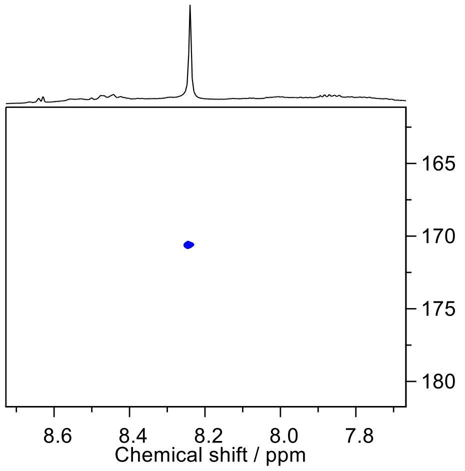

Figure S4. Correlation signal characteristic of formate, observed in the H,C-HSQC spectrum of SF0.

Owing to the sample treatment, formate was observable only in SF0 but not in SF1 since the latter was obtained after HPLC and lyophilization of the acidic solution (containing 0.1% TFA), causing the volatile formic acid to evaporate. We ascribe the presence of formate to the acid-catalyzed reactions of hexoses (glucose, fructose) [1, 2] (being already present or originating from polysaccharide degradation). Other components of this reaction pathway such as hydroxymethylfurfural or levulinic acid have not been found.

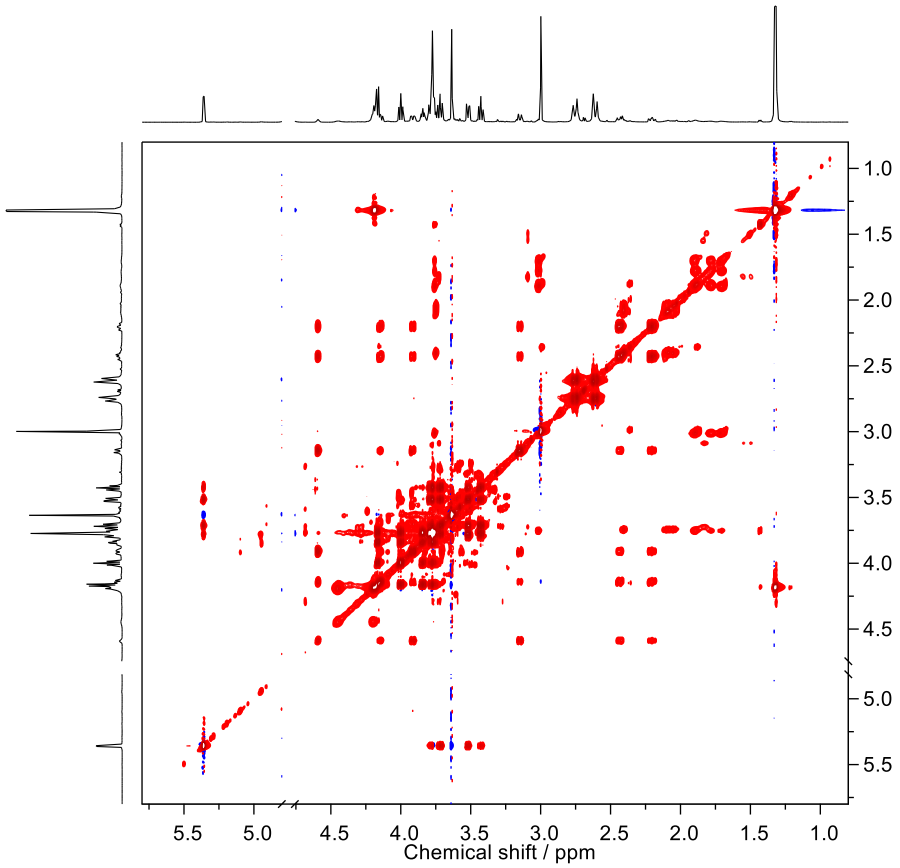

Figure S5. H,H-TOCSY NMR spectrum of SF1 showing the correlations of the main components.

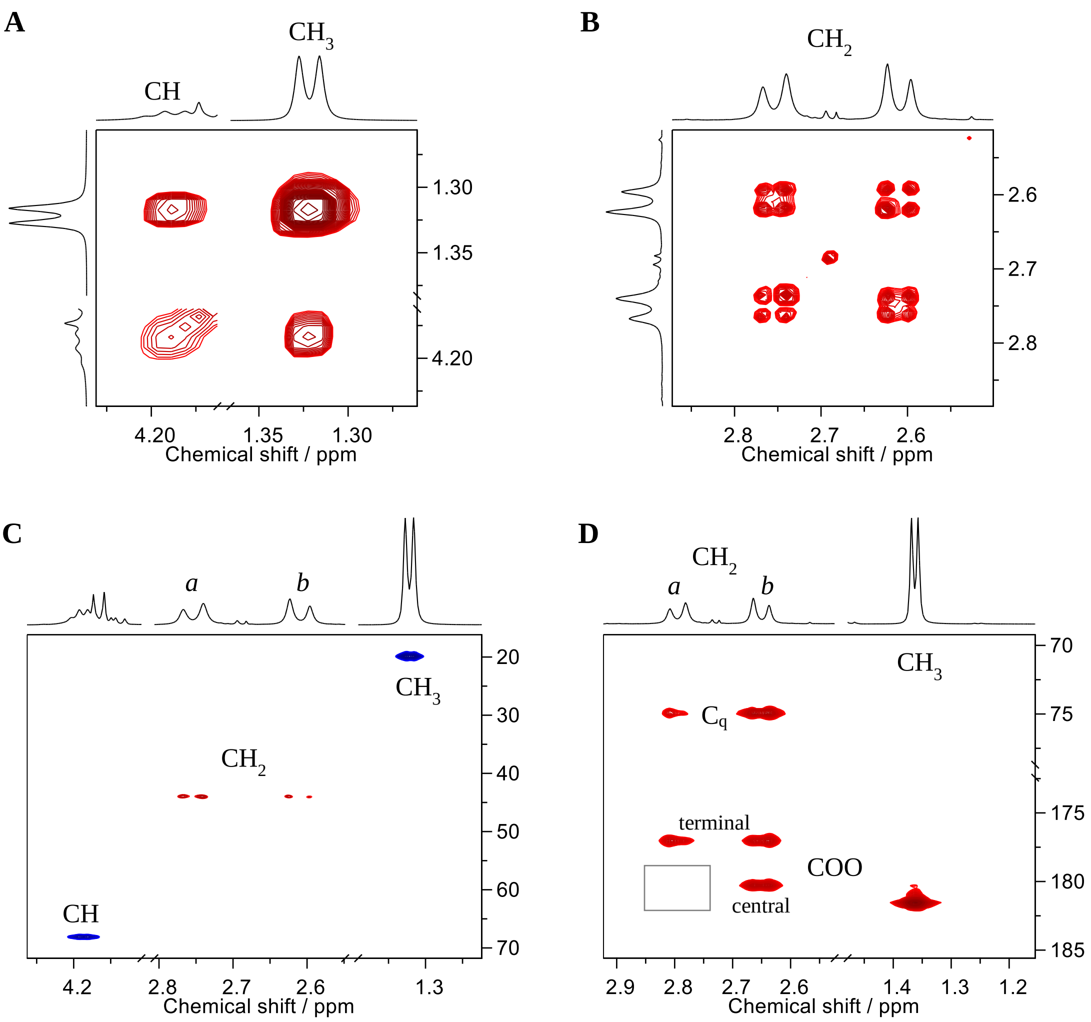

Figure S6. Correlation signals evidencing lactic and citric acids to be present in SF1 as inferred from H,H-TOCSY (A and B, respectively) as well as H,C-HSQC (C) and HMBC spectra (D).

The phase-sensitive HSQC spectrum (C) displays signals due to CH and CH_3 as negative (blue) and CH_2 groups as positive (red). The hydrogens of citrate's methylene groups are diastereotopic (*a* and *b*; but pairwise equivalent). Citrate's central quaternary carbon (C_q) and its two carboxyl groups as well as that of lactate are observable in the HMBC spectrum (D). Owing to the angle-dependence of scalar coupling constants (Karplus relationship) correlations vary in intensity, or even might be not observable as is the case for that between the central carboxyl carbon and H^a (see empty rectangle in D).

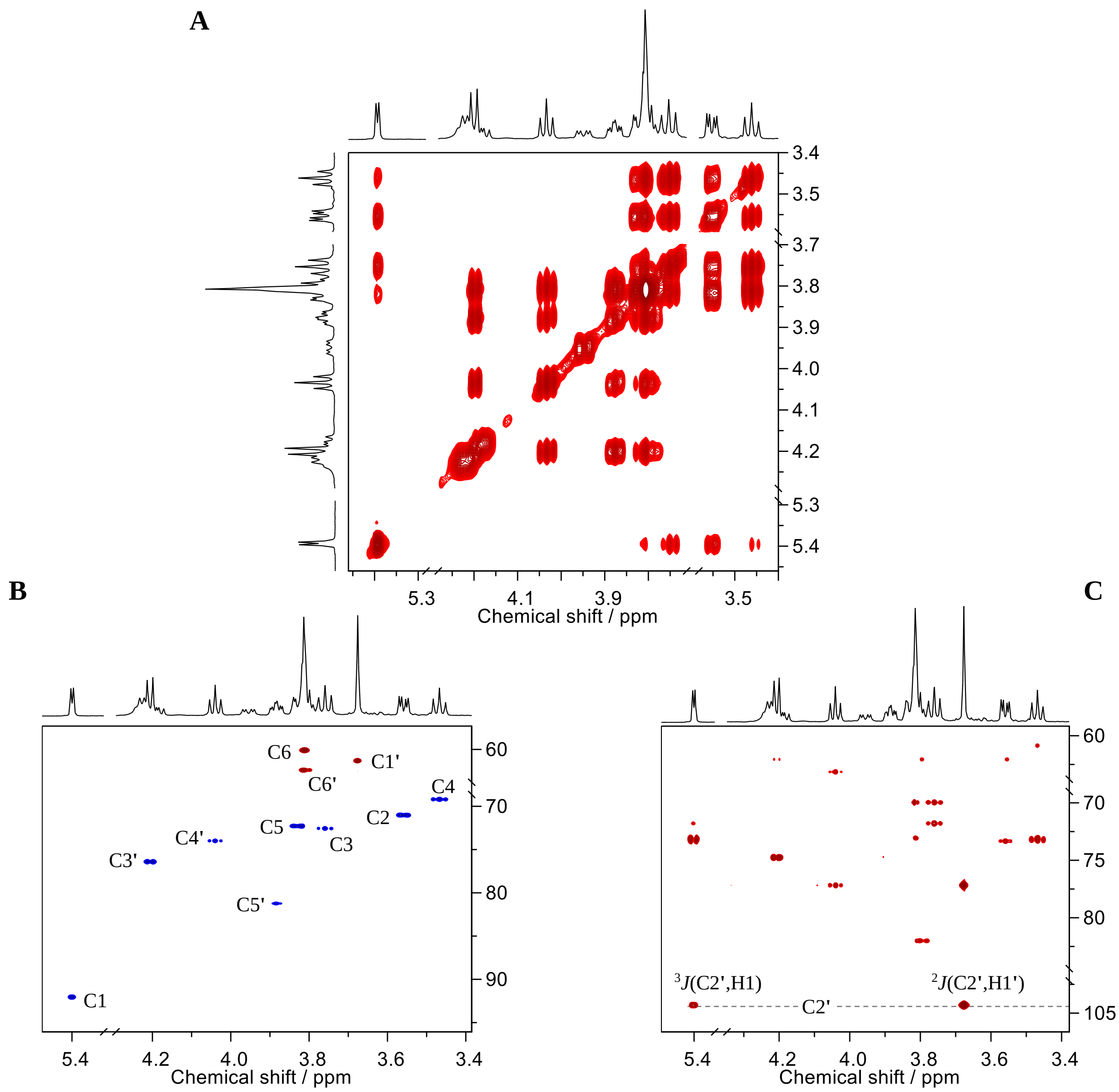

Figure S7. Correlation signals evidencing sucrose to be present in SF1 as inferred from H,H-TOCSY (A) as well as H,C-HSQC (B) and HMBC spectra (C).

Identification of the disaccharide sucrose is straightforward by its characteristic ^{1}H and ^{13}C chemical shifts, especially those involved in the O-α-D-glucopyranosyl-(1→2)-β-D-fructofuranosidic bond, the correlation patterns of the two isolated TOCSY spin systems (A), number and phase of CH and CH_2 HSQC correlation signals (B, CH negative (blue) and CH_2 groups as positive (red)), as well as the long-range C–H correlation between the two sugar units across the glycosidic bond (C).

The high sucrose contents are likely to be the yield of polysaccharide hydrolysis owing to the extraction by aqueous hydrochloric acid.

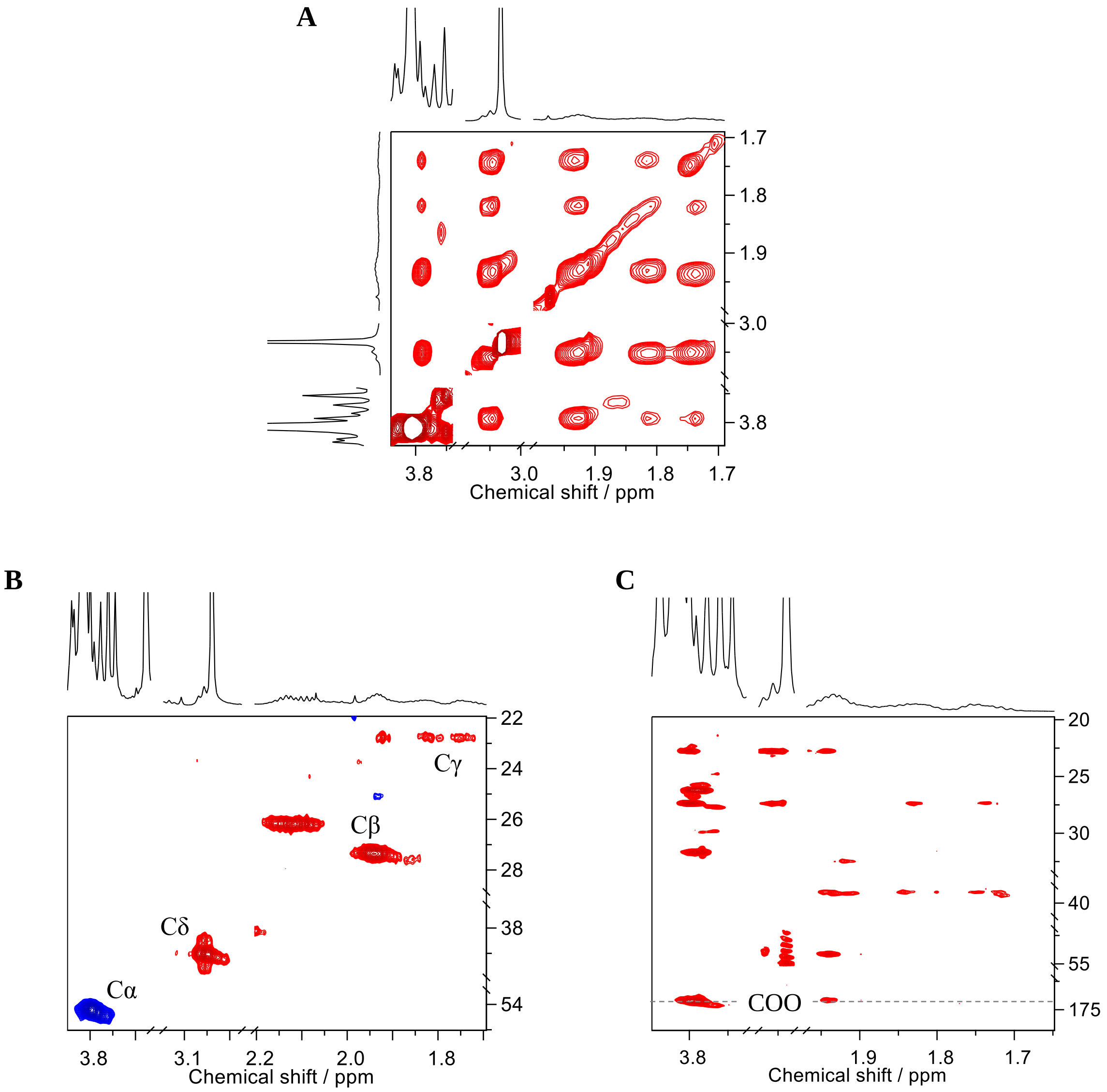

Figure S8. Correlation signals ascribed to ornithine present in SF1 as inferred from H,H-TOCSY (A) as well as H,C-HSQC (B) and HMBC spectra (C).

The TOCSY spectrum (A) reveals scalar coupling all along the entire ^{1}H spin system comprising seven hydrogens. Analogous to the related amino acid lysine, ornithine is short by one methylene group, thus constituting the α-carboxyl group (δ_C 175.0), the H-α (δ_H 3.80), along with the α-amino group and the side chain attached to the C-α (δ_C 54.2), as well as the side chain-terminating amino group at the δ-methylene with characteristic ^{1}H and ^{13}C chemical shifts (3.06 and 39.0 ppm, respectively), and in between three more methylene groups (β, γ, and δ).

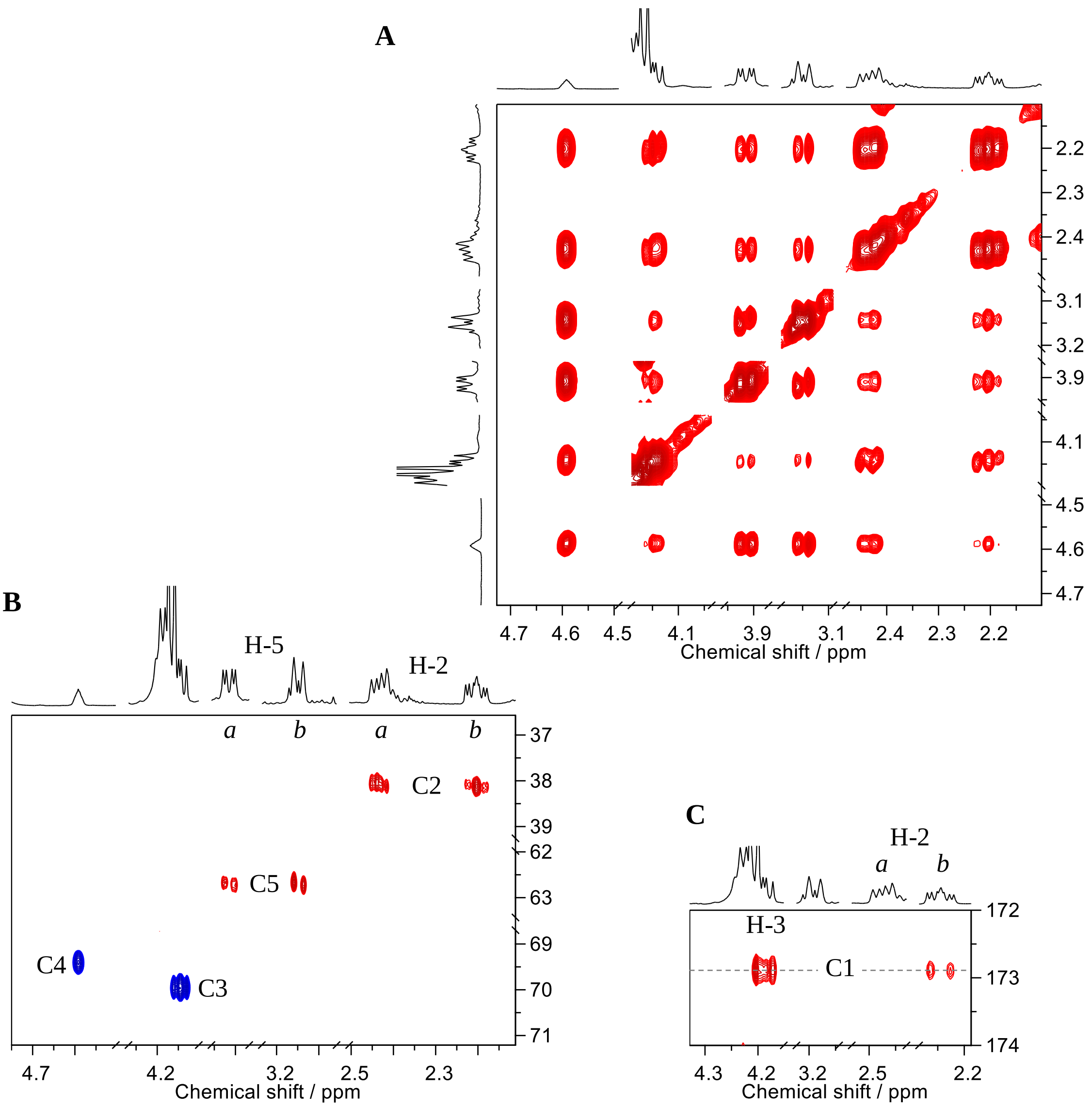

Figure S9. Correlation signals ascribed to a 2-deoxy sugar acid present in SF1 as inferred from H,H-TOCSY (A) as well as H,C-HSQC (B) and HMBC spectra (C).

The TOCSY spectrum (A) reveals scalar coupling all along the entire ^{1}H spin system comprising six distinct hydrogens, two of which resonating significantly upfield (δ_H 2.47 and 2.24 ppm) indicating the deoxygenated carbon which, correspondingly, resonates notably upfield at δ_C 38.4 ppm as seen in the phase-sensitive HSQC spectrum (B) displaying signals due to CH and CH_3 as negative (blue) and CH_2 groups as positive (red). One more methylene group (bearing diastereotopic hydrogens) gives rise to more downfield signals observed at δ_H 3.19 and 3.96 ppm as well as δ_C 62.7 ppm, hence considered to be a CH_2OH residue. We are aware that 3.19 ppm is quite low for such as residue, yet we cannot explain this fact. The two remaining pairs of carbons and hydrogens are due to CHOH groups as inferred from their HSQC signal phase as well as their respective signal positions: δ_H/δ_C with 69.4/4.63 ppm and 70.0/4.19 ppm. Finally, one more carbon is identified in the HMBC spectrum (C): a carboxyl resonating at 172.8 ppm, revealed by the (angle-dependent) ^2J and ^3J couplings to H-2*b* and H-3, respectively.

Upon comparison to literature ^{1}H chemical shifts and coupling constants, 2-deoxyribono-1,4-lactone can be ruled out [3].

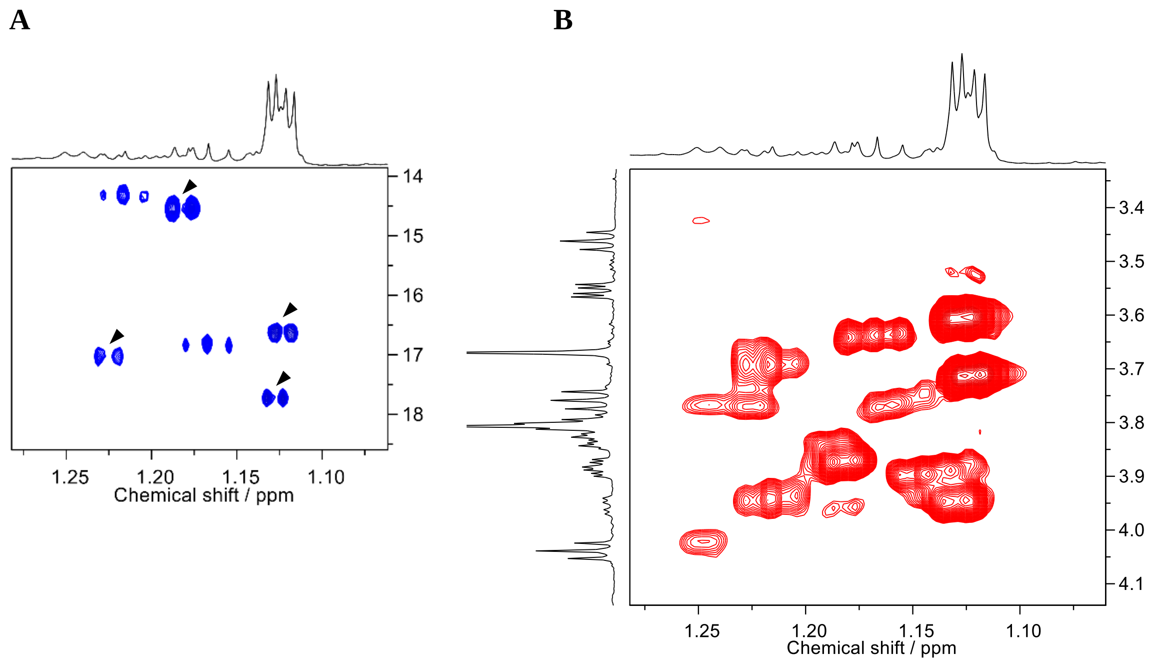

Figure S10. Correlation signals ascribed to 6-deoxy-hexose rhamnose as inferred from H,C-HSQC (A) and H,H-TOCSY spectra (B).

Rhamnose is a common glycone component of glycosides and also found in form of rhamnosides in various nuts, often bound to phenolic compounds or flavonoids [4, 5].

Rhamnose is quite well identified by its methyl group, in the phase-sensitive HSQC spectrum (A) displayed as negative (blue) signals observed in the characteristic (C)–CH_3 ^1H and ^{13}C chemical shift ranges. An additional feature is the split ^1H signal, appearing as a doublet owing to the vicinal coupling (^3J) to H-5 with a coupling constant of ~ (6.2 ± 0.2) Hz, depending on, e.g., whether rhamnose is being linked to another molecule (also impacting conformation) [6-8], as indicated by the spearheads (A). Triplet signals in these spectral regions, accompanied with ^3J about 6.9–7.6 Hz are due to a methyl group bound to a methylene group, (–CH_2CH_3).

In the TOCSY spectrum, the spin coupling is known to be too weak to be distributed throughout the entire spin system comprising H-1 through H-6, even for spin-lock mixing times beyond 80 ms [9]. Therefore, starting from the methyl resonance, often correlations only to H-5 (^3J) and H-4 (^4J) are resolved while the other CH are separated by five or six bonds.

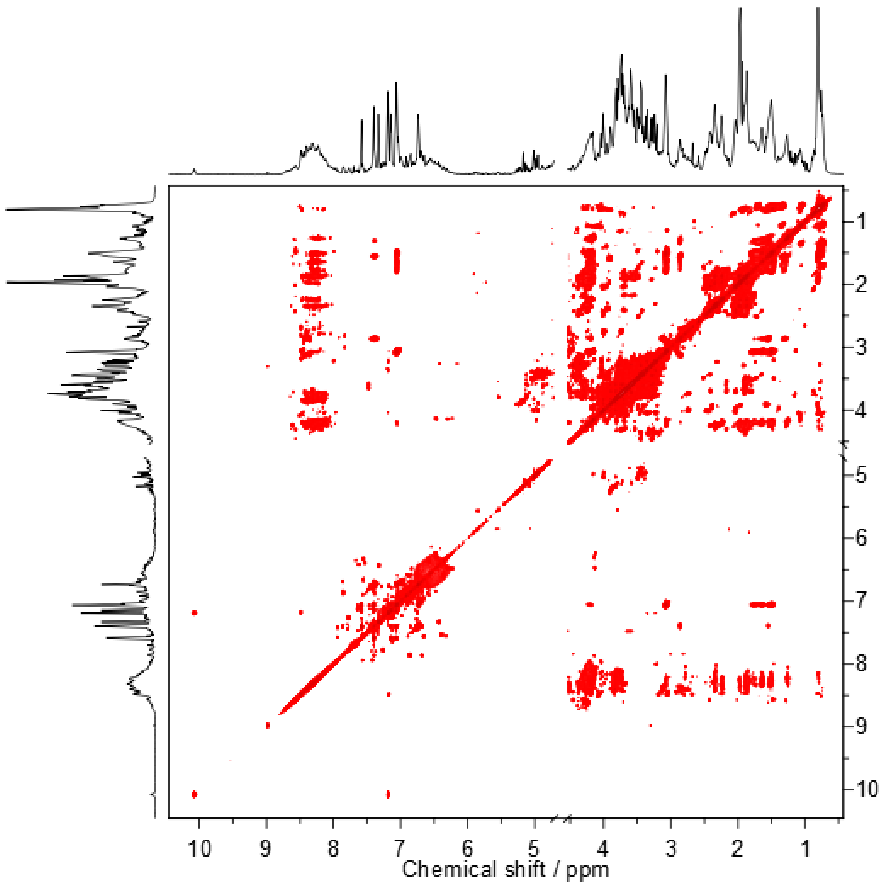

Figure S11. H,H-TOCSY NMR spectrum of SF2.

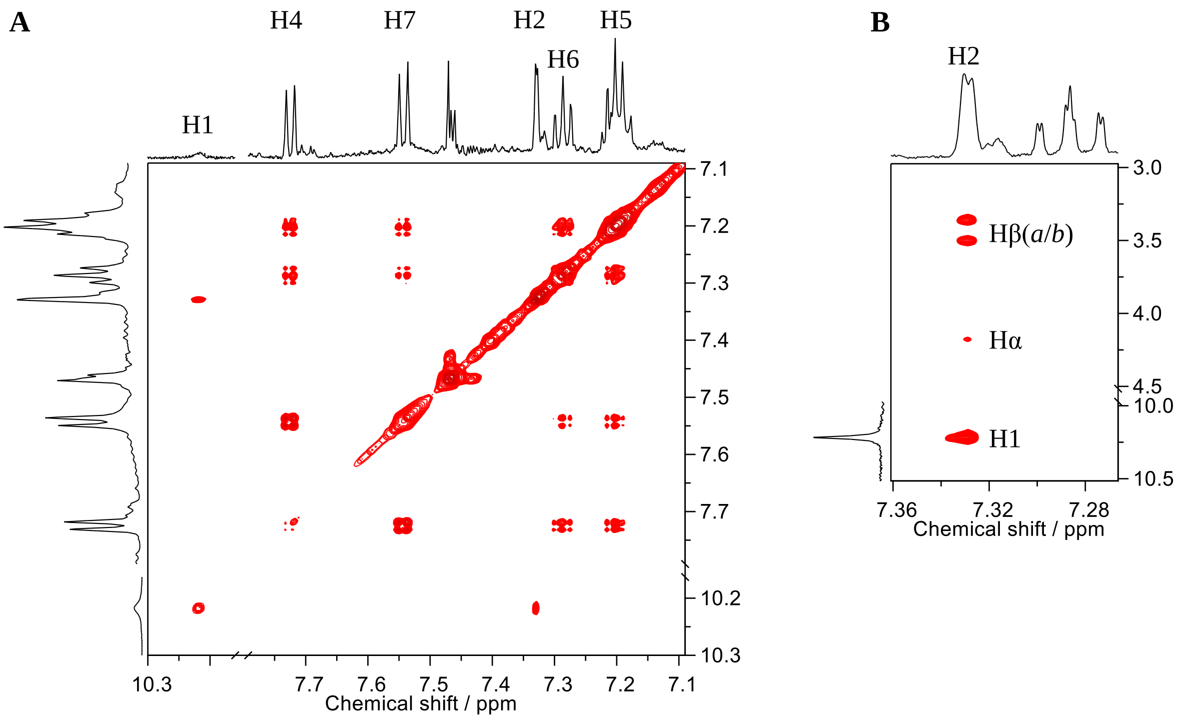

Figure S12 Correlation signals ascribed to tryptophan as inferred from the H,H-TOCSY spectrum.

The two unique spin correlation patterns can unambiguously be assigned to tryptophan. The protons of the six-membered ring (H4 trough H7) constitute an isolated spin system, where two signals are doublets (H4 and H7) and two are triplets (H5 and H6). The N-bound proton of the indole residue (H1) resonates most downfield and in the TOCSY exhibits a correlation only to H2. The latter, however, "connects" the indole's pyrrole spins and the side chain's β and α protons resonating at characteristic chemical shifts [10].

As can be seen from the aromatic region in the ^{1}H NMR spectrum (cf. Figure S4), albeit SF2 is only a few milligrams, it contains tryptophan in notable quantity.

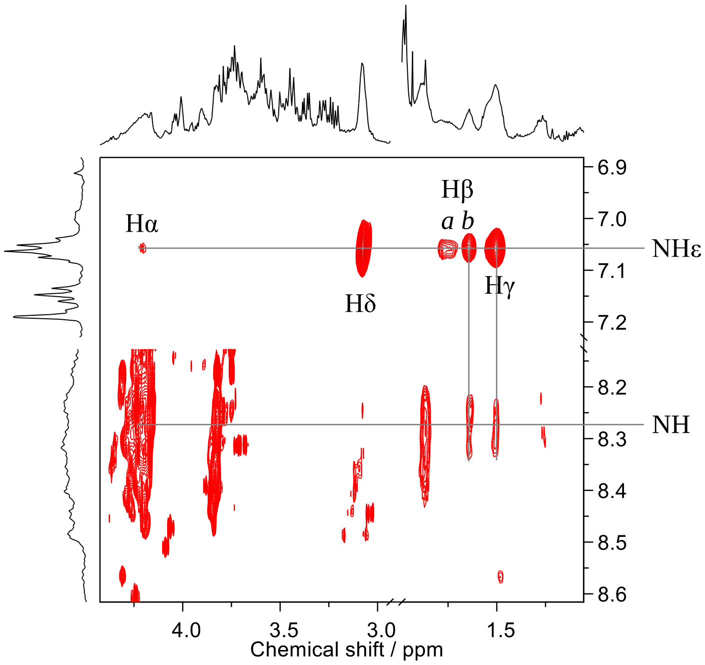

Figure S13. Correlation signals ascribed to peptide-bound arginine/lysine as inferred from the H,H-TOCSY spectrum (SF2).

The tentative assignment of the signals to (peptide-bound) lysine and/or arginine is in fair agreement with literature [10]. The significantly lower chemical shift of the side chain terminal NH (7.06 ppm) compared to those reported for both ARG and LYS (δ_H 8.07 and 7.81, respectively) may be due to some (intra- or intermolecular) interaction with some shielding environment such as the phosphate groups in phytate.

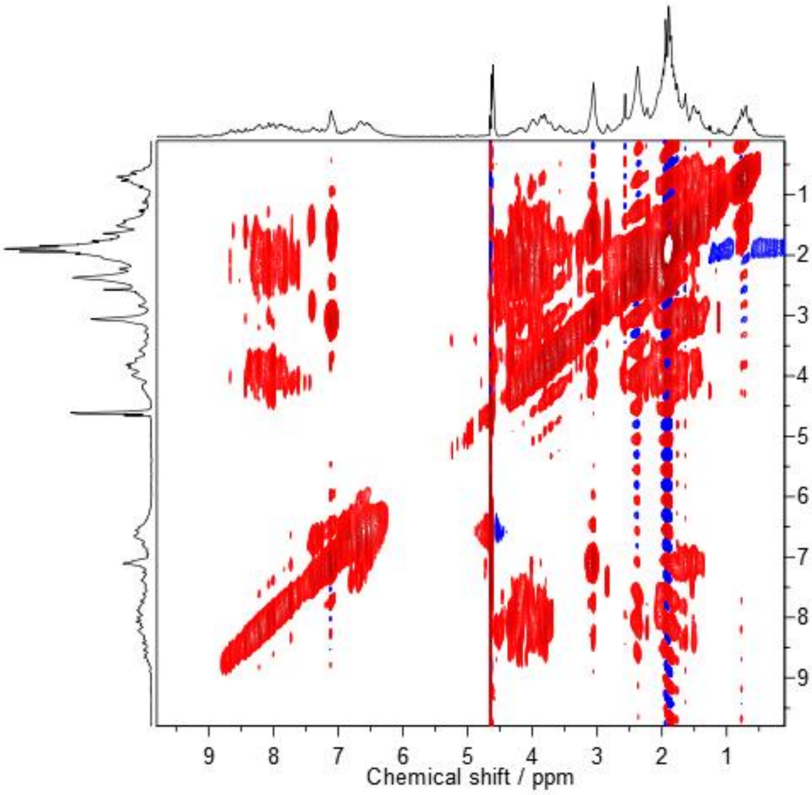

Figure S14. H,H-TOCSY NMR spectrum of SF4.

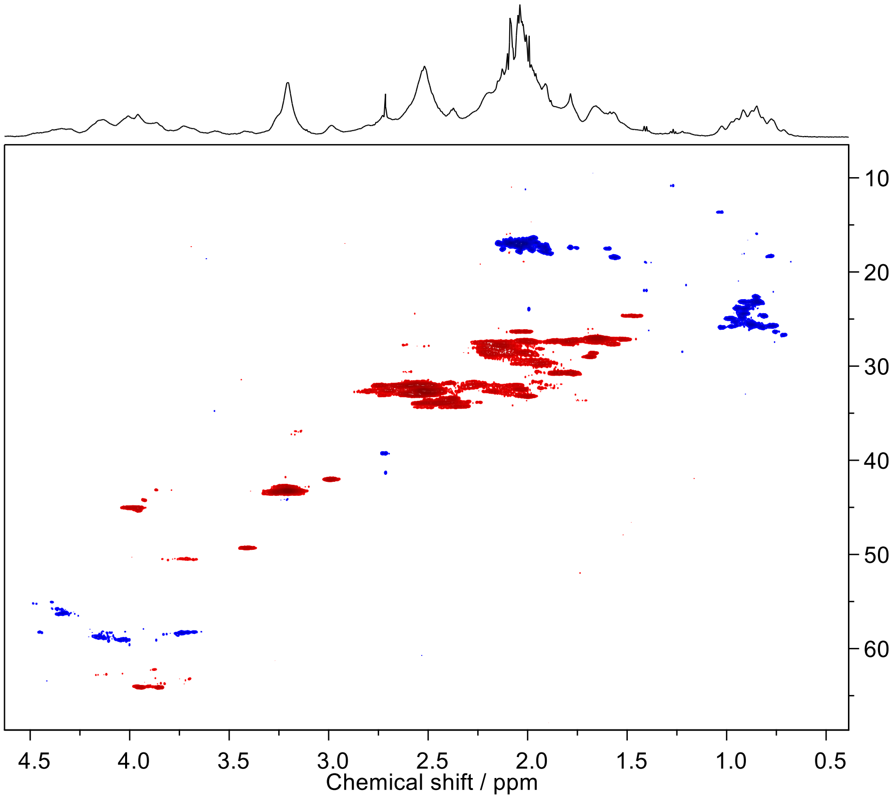

Figure S15. H,C-HSQC NMR spectrum of SF4.

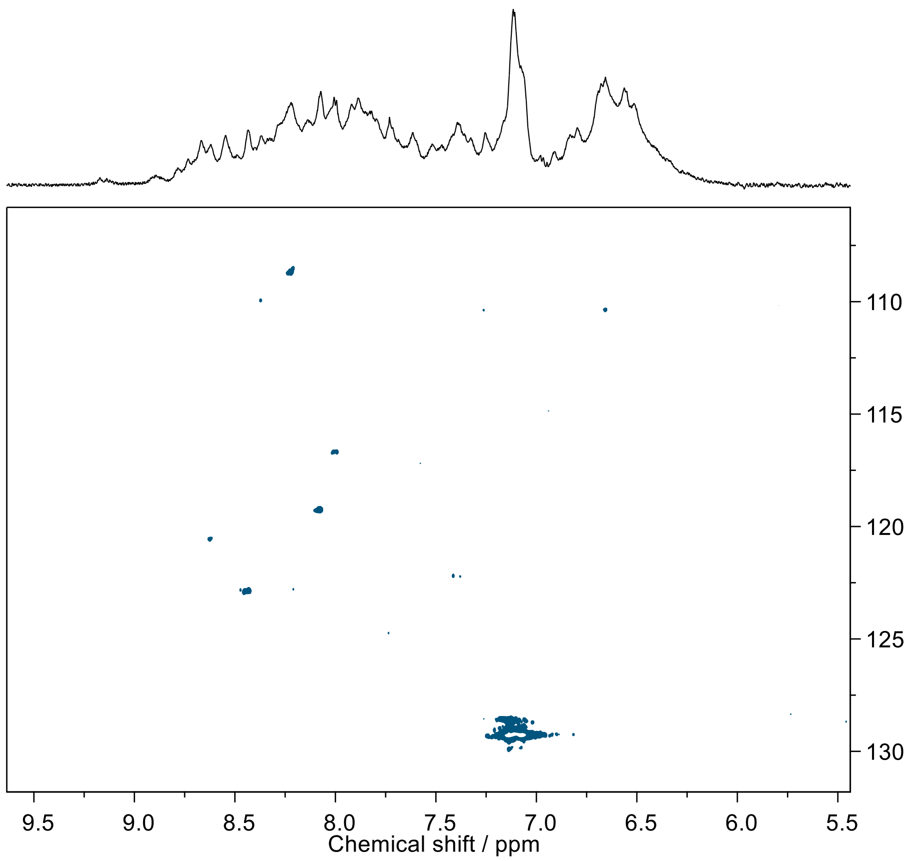

Figure S16. H,N-HSQC NMR spectrum of SF4.

Only a few ^{15}N–^{1}H correlations are detectable. This is mainly due to the low ^{15}N natural abundance of ~0.36% and, owing to the high molecular weight of the molecules, the increased correlation time and thus decreasing transversal relaxation time (T_2), especially affecting ^{1}H nuclei. The signals with δ_N ~110 ppm arise from glycine NH – given their observability and nature – probably residing in some more flexible/mobile residues. The correlation at δ_H/δ_N ~7.1/129 ppm is indicative of lysine ε-NH_3^+ (cf. the considerations stated with Figure S13 and the suspected significantly lower chemical shift of the side chain terminal NH observed at 7.06 ppm).

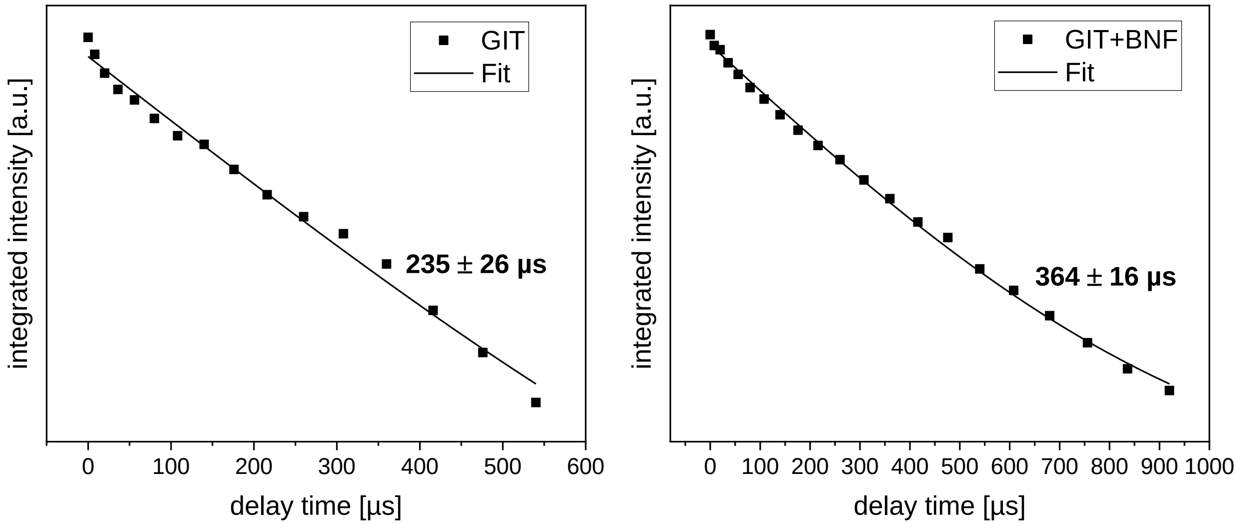

Figure S17. Luminescence decay curves of 10 µM Eu^{3+} in artificial solution of gastrointestinal tract (GIT, left) and with additional brazil nut fluor (BNF, right) at RT and pH = 6.5 ± 0.5 and resulting luminescence lifetimes.

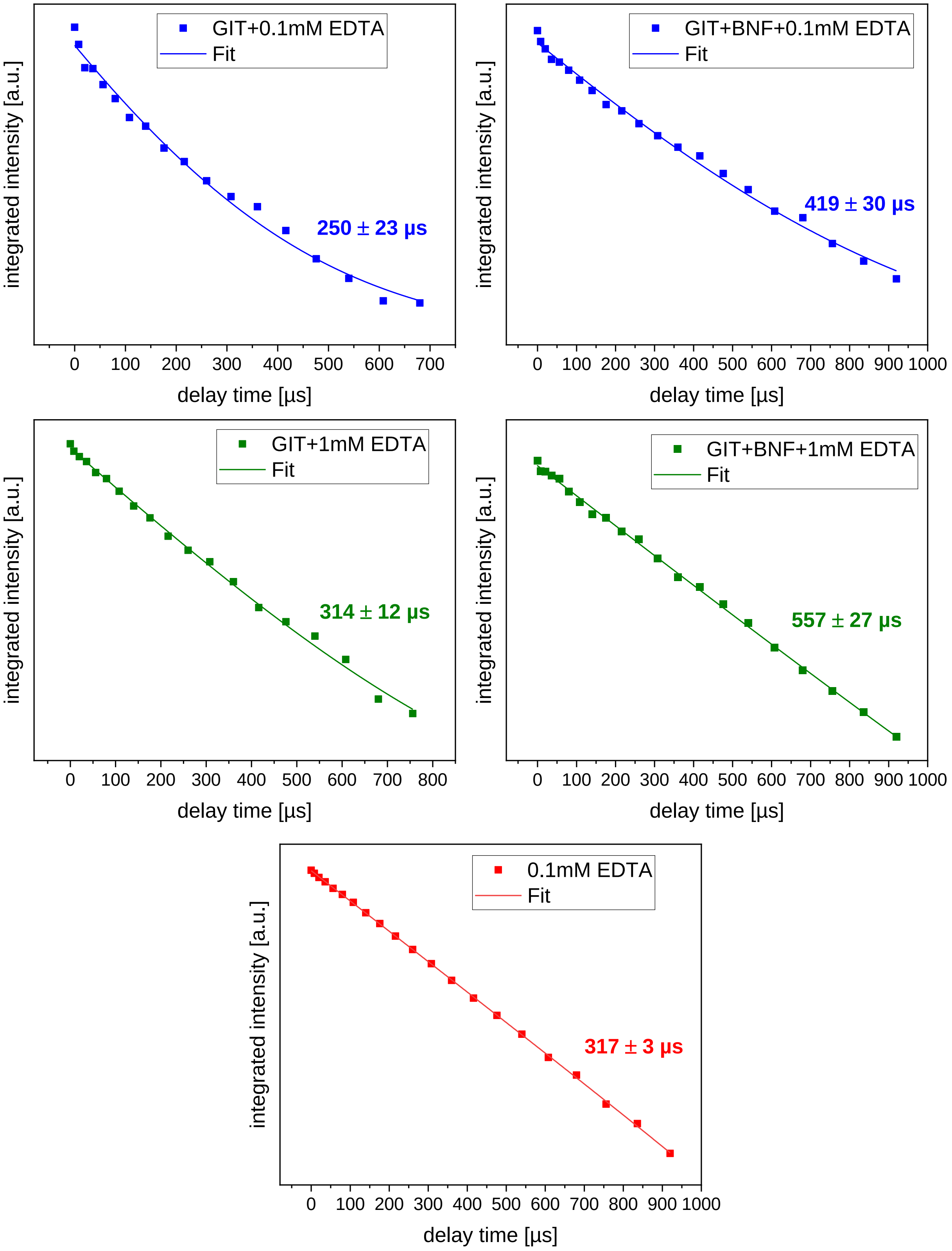

Figure S18. Luminescence decay curves of 10 µM Eu^{3+} in artificial solution of GIT without or with additional BNF and with EDTA at RT and pH = 6.5 ± 0.5 and resulting luminescence lifetimes.

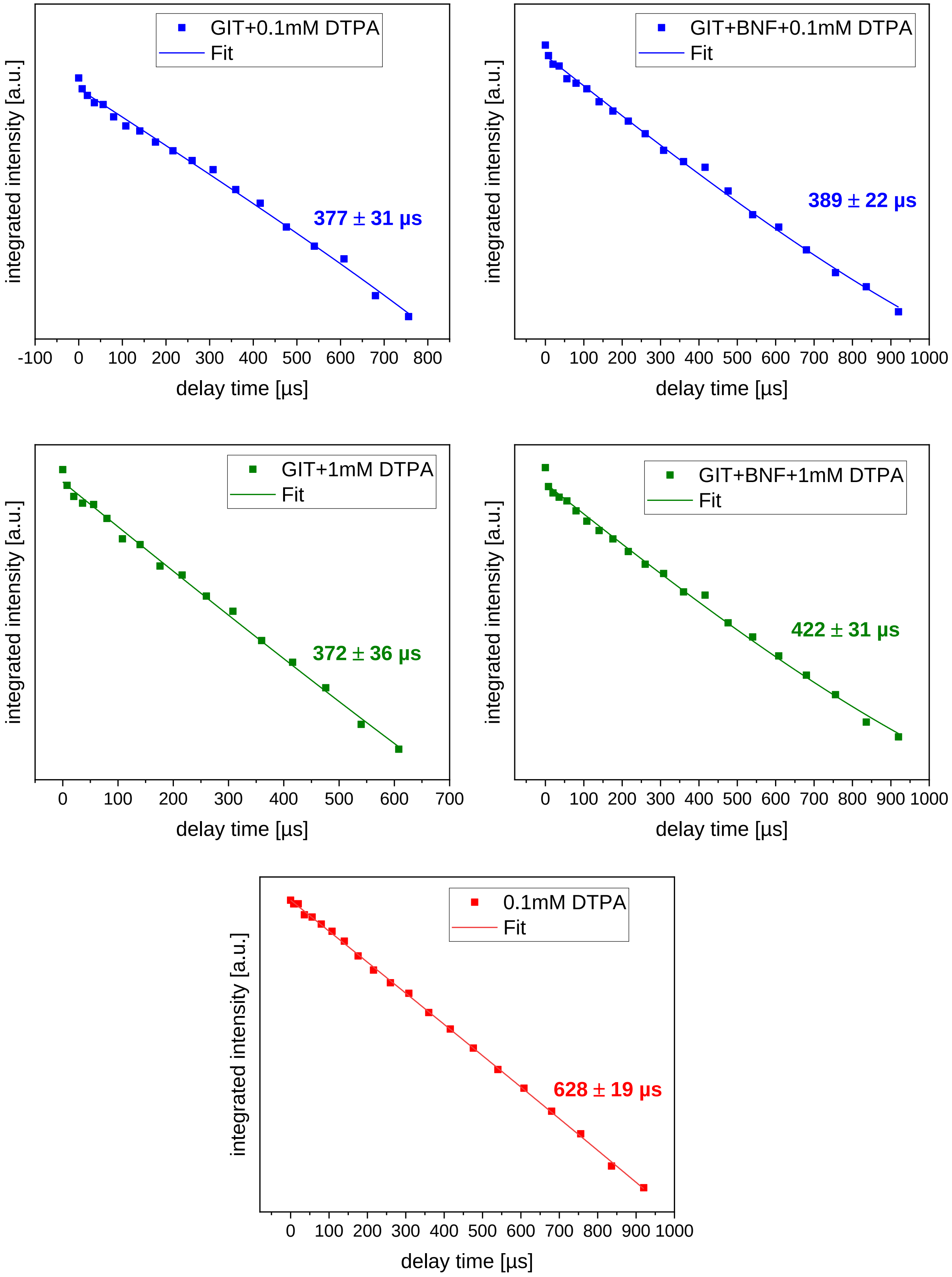

Figure S19. Luminescence decay curves of 10 µM Eu^{3+} in artificial solution of GIT without or with additional BNF and with DTPA at RT and pH = 6.5 ± 0.5 and resulting luminescence lifetimes.

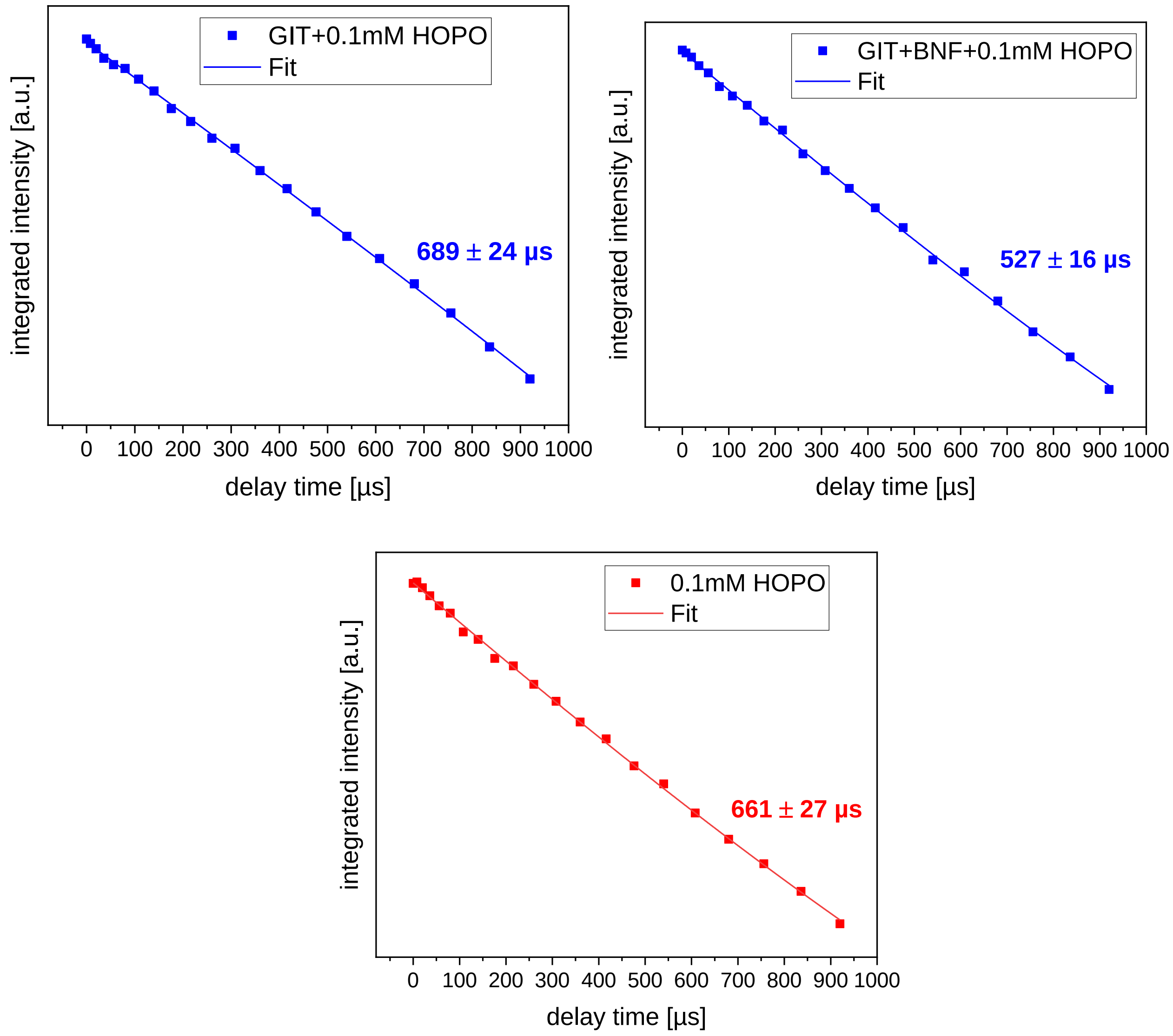

Figure S20. Luminescence decay curves of 10 µM Eu^{3+} in artificial solution of GIT without or with additional BNF and with HOPO at RT and pH = 6.5 ± 0.5 and resulting luminescence lifetimes.

References

1. Li, L.Y., Shen, F., Smith, R.L. and Qi, X.H., *Quantitative chemocatalytic production of lactic acid from glucose under anaerobic conditions at room temperature.* Green Chemistry, 2017. **19**(1): 76-81. DOI: 10.1039/c6gc02443b.
2. Ma, C.Y., Cai, B., Zhang, L., Feng, J.F. and Pan, H., *Acid-Catalyzed Conversion of Cellulose Into Levulinic Acid With Biphasic Solvent System.* Frontiers in Plant Science, 2021. **12**: 10. DOI: 10.3389/fpls.2021.630807.
3. Buchko, G.W. and Cadet, J., *Identification of 2-deoxy-d-ribono-1,4-lactone at the site of benzophenone photosensitized release of guanine in 2'-deoxyguanosine and thymidylyl-(3'-5')-2'-deoxyguanosine.* Canadian Journal of Chemistry-Revue Canadienne De Chimie, 1992. **70**(6): 1827-1832.
4. Bodoira, R. and Maestri, D., *Phenolic Compounds from Nuts: Extraction, Chemical Profiles, and Bioactivity.* Journal of Agricultural and Food Chemistry, 2020. **68**(4): 927-942. DOI: 10.1021/acs.jafc.9b07160.
5. Ojeda-Amador, R.M., Salvador, M.D., Fregapane, G. and Gómez-Alonso, S., *Comprehensive Study of the Phenolic Compound Profile and Antioxidant Activity of Eight Pistachio Cultivars and Their Residual Cakes and Virgin Oils.* Journal of Agricultural and Food Chemistry, 2019. **67**(13): 3583-3594. DOI: 10.1021/acs.jafc.8b06509.
6. Lipkind, G.M., Nifantev, N.E., Shashkov, A.S. and Kochetkov, N.K., *NMR and conformational study of branched oligosaccharides containing 2,3-disubstituted residues of alpha-l-rhamnose.* Canadian Journal of Chemistry, 1990. **68**(7): 1238-1250.
7. Colson, P. and King, R.R., *C-13-NMR spectra of disaccharides of d-glucose, d-galactose, and l-rhamnose as models for immunological polysaccharides.* Carbohydrate Research, 1976. **47**(1): 1-13.
8. Debruyn, A., Anteunis, M., Degussem, R. and Dutton, G.G.S., *H-1-NMR study of l-rhamnose, methyl alpha-l-rhamnopyranoside, and 4-o-beta-d-galactopyranosyl-l-rhamnose in deuterium-oxide.* Carbohydrate Research, 1976. **47**(1): 158-163.
9. Gheysen, K., Mihai, C., Conrath, K. and Martins, J.C., *Rapid Identification of Common Hexapyranose Monosaccharide Units by a Simple TOCSY Matching Approach.* Chemistry-a European Journal, 2008. **14**(29): 8869-8878. DOI: 10.1002/chem.200801081.
10. Wishart, D.S., Bigam, C.G., Holm, A., Hodges, R.S. and Sykes, B.D., *H-1, C-13 and N-15 random coil nmr chemical-shifts of the common amino-acids .1. Investigations of nearest-neighbor effects.* Journal of Biomolecular NMR, 1995. **5**(1): 67-81.
